# SHP-1 phosphatase acts as a coactivator of *PCK1* transcription to control gluconeogenesis

**DOI:** 10.1016/j.jbc.2023.105164

**Published:** 2023-08-16

**Authors:** Amit Kumar, Michael Schwab, Beisy Laborit Labrada, Maruhen Amir Datsch Silveira, Marilyn Goudreault, Éric Fournier, Kerstin Bellmann, Nicole Beauchemin, Anne-Claude Gingras, Steve Bilodeau, Mathieu Laplante, André Marette

**Affiliations:** 1 Faculté de Médecine, Centre de recherche de l'Institut universitaire de cardiologie et de pneumologie de Québec (CRIUCPQ), Université Laval, Québec, Quebec, Canada; 2 Centre de Recherche du CHU de Québec - Université Laval, Axe Oncologie, Québec, Quebec, Canada; 3 Centre de Recherche sur le Cancer de l’Université Laval, Québec, Quebec, Canada; 4 Département de biologie moléculaire, biochimie médicale et pathologie, Faculté de Médecine, Université Laval, Québec, Quebec, Canada; 5 Institute for Research in Immunology and Cancer, Université de Montréal, Montréal, Quebec, Canada; 6 Lunenfeld-Tanenbaum Research Institute, Mount Sinai Hospital, Sinai Health System, Toronto, Ontario, Canada; 7 Centre de recherche en données massives de l’Université Laval, Québec, Quebec, Canada; 8 Department of Oncology, Medicine and Biochemistry, Rosalind and Morris Goodman Cancer Institute, McGill University, Montreal, Quebec, Canada; 9 Department of Molecular Genetics, University of Toronto, Toronto, Ontario, Canada; 10 Institute of Nutrition and Functional Foods, Laval University, Québec, Quebec, Canada

**Keywords:** SHP-1/PTPN6, PCK1, gluconeogenesis, transcription, liver

## Abstract

We previously reported that the protein-tyrosine phosphatase SHP-1 (PTPN6) negatively regulates insulin signaling, but its impact on hepatic glucose metabolism and systemic glucose control remains poorly understood. Here, we use co-immunoprecipitation assays, chromatin immunoprecipitation sequencing, *in silico* methods, and gluconeogenesis assay, and found a new mechanism whereby SHP-1 acts as a coactivator for transcription of the phosphoenolpyruvate carboxykinase 1 (*PCK1*) gene to increase liver gluconeogenesis. SHP-1 is recruited to the regulatory regions of the *PCK1* gene and interacts with RNA polymerase II. The recruitment of SHP-1 to chromatin is dependent on its association with the transcription factor signal transducer and activator of transcription 5 (STAT5). Loss of SHP-1 as well as STAT5 decrease RNA polymerase II recruitment to the *PCK1* promoter and consequently *PCK1* mRNA levels leading to blunted gluconeogenesis. This work highlights a novel nuclear role of SHP-1 as a key transcriptional regulator of hepatic gluconeogenesis adding a new mechanism to the repertoire of SHP-1 functions in metabolic control.

SHP-1 (encoded by gene *PTPN6*) is a nonmembrane protein tyrosine phosphatase (PTP) that plays important roles in controlling immune signaling pathways and fundamental physiological processes (1, 2, 3, 4, 5). SHP-1 encompasses two SH2 domains at the N terminus followed by a central phosphatase domain and a C-terminal regulatory tail (6, 7). SHP-1 mainly exerts its functions by dephosphorylating target proteins in diverse signaling pathways in the cytoplasm, but is also found in the nucleus of epithelial cells (8, 9, 10). Although the function of the SHP-1 nuclear pool remains mostly elusive, it has been reported that SHP-1 negatively regulates the activity of some transcription factors by dephosphorylation (11, 12).

While SHP-1 is predominantly expressed in hematopoietic cells of all lineages, we have shown that this phosphatase is also expressed at lower levels in cells of metabolically active tissues such as the liver, skeletal muscle, and adipose tissue, where its expression is increased in obese mice fed a high-fat diet (3). We first reported that SHP-1 negatively regulates glucose metabolism and insulin action through interfering with the insulin receptor-phosphoinositide 3-kinase-AKT (IR-PI3K-AKT) axis, and by inhibiting insulin clearance targeting carcinoembryonic antigen related cell adhesion molecule 1 (CEACAM1) thus ultimately contributing to obesity-linked insulin resistance (4, 13, 14). Pharmacologic inhibition and siRNA-mediated SHP-1 downregulation in diet-induced obese mice improve insulin sensitivity and glucose tolerance (15). However, SHP-1 also controls liver metabolism and glucose homeostasis independently from its ability to impede insulin signaling as shown by the lower fasting glucose and markedly decreased hepatic glucose production in mice with hepatocyte-specific conditional deletion of SHP-1 (*Ptpn6*^*H-KO*^) than their WT (*Ptpn6*^*f/f*^) littermates (3). The underlying mechanism(s) involved in this liver phenotype remain to be elucidated.

Gluconeogenesis is a process by which the liver can synthesize glucose from noncarbohydrate sources during starvation (16), which can contribute to hyperglycemia in type 2 diabetic patients when not tightly controlled, resulting in increased endogenous glucose production (17). Phosphoenolpyruvate carboxykinase 1 (PCK1/PEPCK) is the key rate-limiting enzyme controlling gluconeogenesis (18, 19). PCK1 catalyzes the conversion of oxaloacetate to phosphoenolpyruvate. *Pck1* gene silencing in mouse liver resulted in improved glycemic control and insulin sensitivity (20) whereas higher levels of *PCK1* transcripts have been reported in diabetic patients (21). PCK1 activity is largely correlated with its transcription, which is controlled by several transcription factors and coregulators, in turn regulated by hormones and diet (22, 23, 24). Therefore, it is important to fully understand the mechanistic details of *PCK1* regulation at the transcriptional level, since altering hepatic gluconeogenesis by modulating *PCK1* gene expression could be a therapeutic approach to treat diabetes.

Both the nuclear and metabolic function of SHP-1 have been poorly characterized. Here, we describe the RNA polymerase II (RNA pol II) subunit POLR2J as a novel interaction partner of SHP-1. We demonstrate a novel function of nuclear SHP-1 by acting as a transcriptional coactivator regulating *PCK1* gene expression. We show that SHP-1 and the transcription factor signal transducer and activator of transcription 5 (STAT5) are interdependently associated with chromatin to control recruitment of RNA pol II to the *PCK1* promoter. Loss of SHP-1 as well as STAT5 inactivation or depletion decreased *PCK1* transcription and consequently gluconeogenesis in hepatocytes. In this study, we have demonstrated a new nuclear mechanism for SHP-1 by which it regulates *PCK1*-transcription and hepatic glucose production.

## Results

### SHP-1 interacts with proteins of the transcriptional machinery

**Figure 1:**
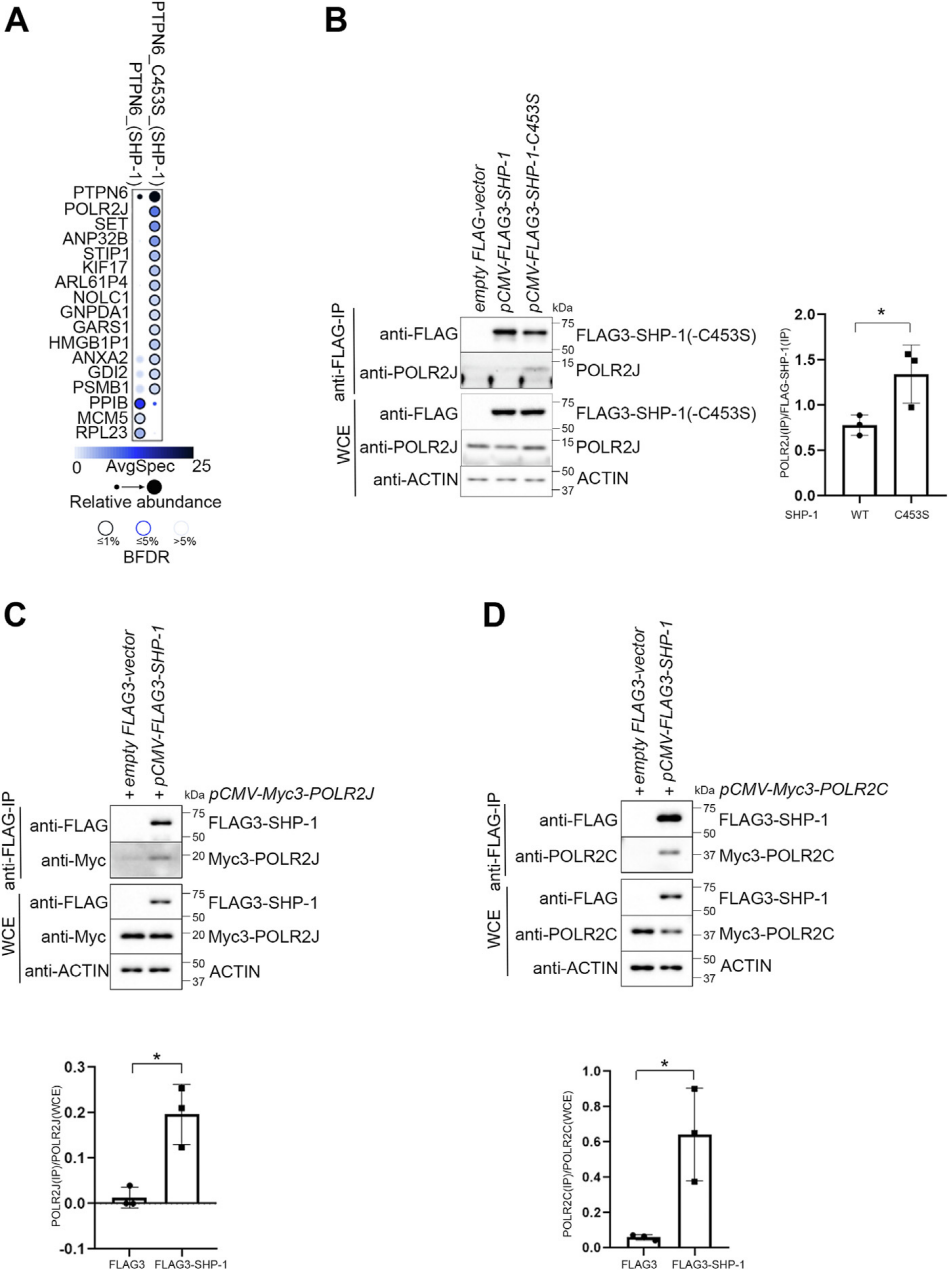
**SHP-1 interacts with proteins of the transcriptional machinery.***A*, dot plot of the confident interactions identified by mass spectrometry after co-immunoprecipitation with SHP-1 in Flp-In T-REx 293 cells. The color-coding maps to average spectral counts (capped at 25), and the size of the circle to the relative spectral abundance across the baits. The *edge color* represents the Bayesian false discovery rate (BFDR); see legend inset for details. *B*, Western blot analysis of co-immunoprecipitation showing binding of inducibly expressed FLAG3-SHP-1 constructs to endogenous POLR2J in Flp-In T-REx 293 cells. Quantification of POLR2J/SHP-1 binding determined by densitometry using ImageJ; ∗*p* < 0.05 (n = 3). *C* and *D*, co-immunoprecipitation experiments showing the association of transiently expressed FLAG3-SHP-1 with Myc3-POLR2J and Myc3-POLR2C in HepG2 cells. Quantification of coprecipitated POLR2J and POLR2C determined by densitometry using ImageJ; ∗*p* < 0.05 (n = 3). WCE, whole cell extract.

To characterize new, regulatory metabolic SHP-1-functions, we sought to identify SHP-1-interacting partners using an affinity-purification mass spectrometry approach (25). We generated stably transfected Flp-In 293 T-REx cells, which inducibly express proteins of interest from a common locus. Total extracts from cells producing FLAG-tagged WT SHP-1 or the SHP-1-C453S substrate-trapping mutant, which was shown to stabilize the association between phosphatase and substrate (26), were FLAG-immunoprecipitated. The resulting samples were analyzed by liquid chromatography-tandem mass spectrometry. To define specific interactors of SHP-1, cells expressing an empty FLAG-vector and a fusion of FLAG to the GFP, described elsewhere (27) were used as controls. SAINTexpress (28) was used to identify the high-confidence interaction partners for SHP-1 or SHP-1-C453S (Table S1). The WT and C453S constructs recovered largely different sets of interaction partners, with the C453S mutant recovering more interaction partners. Interestingly, POLR2J (RPB11), a small subunit of RNA pol II, was identified as one of the novel significant SHP-1-C453-associated proteins after FLAG-purification mass spectrometry (Fig. 1*A* and Table S1). Co-immunoprecipitation (co-IP) experiments in the Flp-In 293 T-REx cells confirmed the binding of POLR2J to SHP-1 and, consistent with the mass spectrometry analysis, showed that endogenous POLR2J interacted mainly with the substrate-trapping SHP-1-C453S mutant (Fig. 1*B*). To validate the association between SHP-1 and POLR2J we performed co-IP experiments in HepG2 hepatic cells. We confirmed that exogenously coexpressed FLAG-tagged SHP-1 interacted with Myc-tagged POLR2J in these cells (Fig. 1*C*) and that SHP-1 was also bound to POLR2C (RPB3), another subunit of RNA pol II closely associated with POLR2J (Fig. 1*D*).

To evaluate, whether the active site of SHP-1 plays a major role in the recognition of POLR2J, as suggested by the increased binding of POLR2J to the SHP-1-C453S substrate trapping mutant, we tested the interaction of POLR2J with several, catalytically inactive mutants of SHP-1 (26, 29) (Fig. S1). Whereas the substrate-trapping mutant C453S showed a better interaction with POLR2J than WT SHP-1, the binding of another substrate-trapping mutant D419A to POLR2J was not increased. While the R459M- and A455T-mutants, which render SHP-1 catalytically inactive or less active, respectively, were still able to bind to POLR2J to a similar extent as WT SHP-1, a mutant with a deleted active site (ΔP, residues 451–475 deleted) interacted better with POLR2J. Because these data indicate that another important part of SHP1 besides the catalytic domain exists, we tested truncation constructs of SHP-1 for their association with POLR2J (Fig. S2). Although a C-terminal fragment containing the PTPase domain still interacted with POLR2J, an N-terminal fragment carrying the two SH2-domains bound better to POLR2J. This suggests the existence of either two binding sites on POLR2J, which interact with different sites of SHP-1, or a single binding site, which binds to the SH2-domains or the catalytic domain of SHP-1 with different affinities.

**Figure 2:**
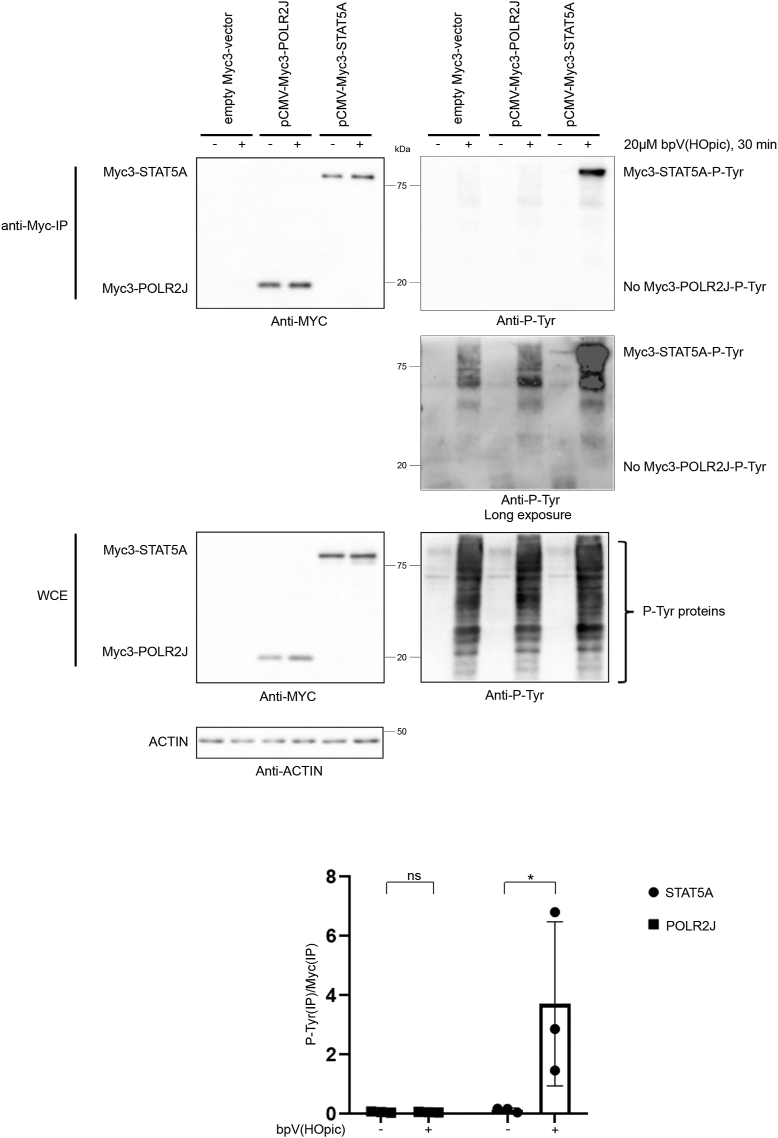
**STAT5, but not POLR2J is tyrosine-phosphorylated.** Western blot analysis of immunoprecipitations of Myc-tagged proteins showing tyrosine phosphorylation of STAT5A, but not of POLR2J. HepG2-cells were transfected with an empty Myc3-vector, Myc3-POLR2J- or Myc3-STAT5A-expressing plasmids and treated with 20 μM bpV(HOpic) for 30 min or left untreated. Tyrosine-phosphorylation of respective Myc3-tagged proteins precipitated with anti-Myc agarose beads was analyzed in immunoblots with phospho-tyrosine-specific antibodies. Quantification of phospho-tyrosine blot of Myc-precipitated samples determined by densitometry using ImageJ; ∗*p* < 0.05, ns = nonsignificant (n = 3). STAT5, signal transducer and activator of transcription 5; WCE, whole cell extract.

The interaction of POLR2J with the SH2-domains of SHP-1, which are phospho-tyrosine recognition domains, as well as the increased binding of POLR2J to the substrate-trapping SHP-1-C453S mutant imply that POLR2J should be tyrosine-phosphorylated to be recognized by SHP-1 as target. However, we were not able to detect any tyrosine-phosphorylation of POLR2J in a series of experiments. Immunoprecipitated POLR2J from HepG2-cells treated with the potent, general tyrosine phosphatase inhibitor bpV(HOpic) did not show any tyrosine phosphorylation signal, whereas the tyrosine phosphorylation of the transcription factor STAT5A, a well-known tyrosine phosphorylated protein, was easily detectable under the same conditions (Fig. 2). In two other similar experiments, neither cooverexpression of the dominant-negative SHP-1-C453S mutant alone or in combination with bpV(HOpic)-treatment were able to induce tyrosine-phosphorylation of POLR2J, although the SHP-1 construct itself was readily tyrosine-phosphorylated in the same cells after bpV(HOpic)-treatment (Fig. S3, *A* and *B*). This suggests that the interaction between SHP-1 and POLR2J is independent of tyrosine phosphorylation and that POLR2J is not a direct substrate of SHP-1.

Together, these data demonstrate a complex, physical interaction between SHP-1 and RNA pol II in hepatic cells suggesting a role for SHP-1 in transcriptional regulation.

### SHP-1 localizes to the chromatin-bound nuclear fraction

**Figure 3:**
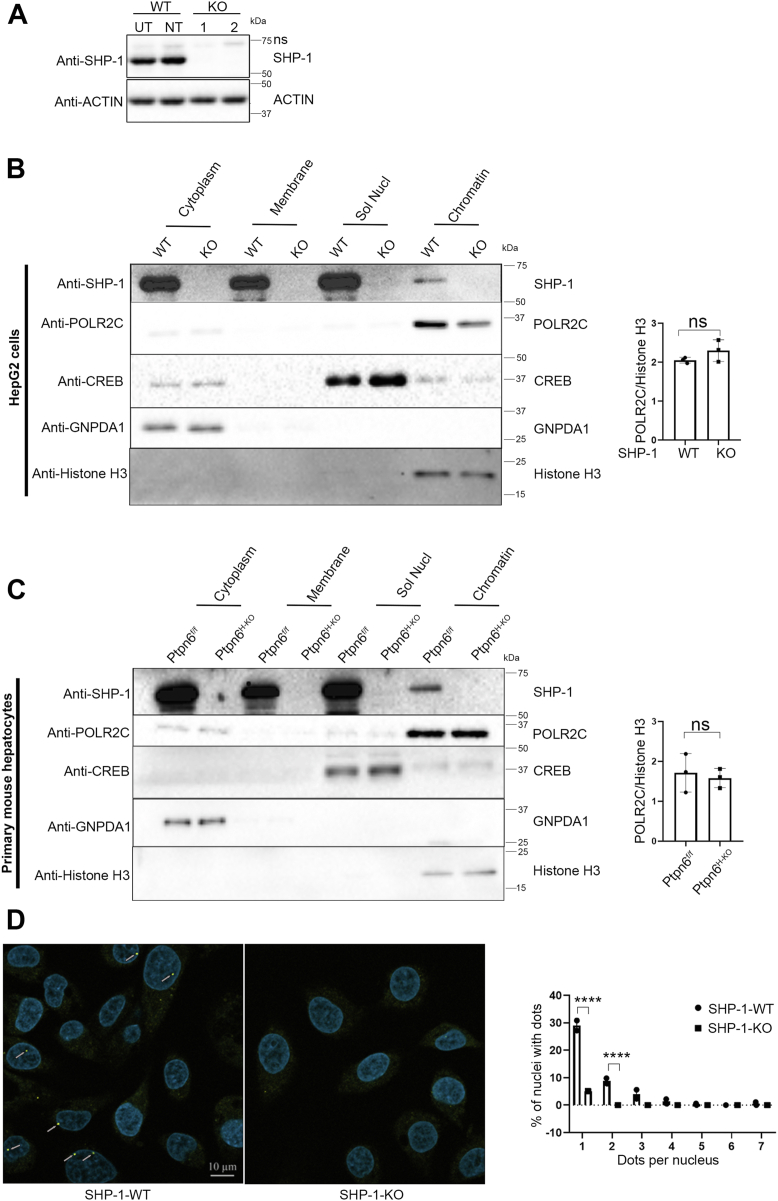
**SHP-1 localizes to the chromatin bound nuclear fraction and interacts endogenously with RNA pol II.***A*, representative Western blot confirming CRISPR-mediated SHP-1 KO in HepG2 cells (UT, untransfected, NT, nontargeting construct, KO, SHP-1 targeting constructs), ns, nonspecific band. *B*, Western blot of HepG2 cell-fractionation assay showing the presence of SHP-1 in cytosolic, membrane, soluble nuclear and chromatin-bound nuclear fraction. Marker proteins confirming enrichment of different fractions. Quantification of POLR2C with histone H3 as loading control in the chromatin fraction determined by densitometry using ImageJ; ns = nonsignificant (n = 3). *C*, Western blot of PMH isolated from *Ptpn6*^*f/f*^ and *Ptpn6*^*H-KO*^ mice cell-fractionation assay showing the presence of SHP-1 in cytosolic, membrane, soluble nuclear, and chromatin-bound nuclear fraction. Marker proteins confirming enrichment of different fractions. Quantification of POLR2C with histone H3 as loading control in the chromatin fraction determined by densitometry using ImageJ; ns = nonsignificant (n = 3). *D*, proximity ligation assay (PLA) with SHP-1- and RPB1-specific antibodies showing colocalization (*yellow dots*, marked with *arrows*) of SHP-1 with RNA pol II in nuclei (DAPI; blue staining) of SHP-1-WT HepG2-cells. Nuclear dots were quantified from at least 400 nuclei (n = 2). ∗∗∗∗*p* <0.0001. CRISPR, clustered regularly interspaced short palindromic repeats; DAPI, 4′,6-diamidino-2-phenylindole; PMH, primary mouse hepatocytes; RNA pol II, RNA polymerase II.

SHP-1 exhibits a different intracellular distribution depending on the cell type with a mainly cytoplasmic localization in hematopoietic cells, but predominantly nuclear staining in non-hematopoietic cells (8, 9, 10). To study the localization and function of SHP-1 in hepatocytes in more detail, we generated clonal HepG2 cell lines with and without deletion of *PTPN6* by using CRISPR/Cas9 technology. The KO was confirmed by Western blot (Figs. 3*A* and S4). Since SHP-1 would require to be localized on the chromatin in order to regulate RNA pol II, we performed subcellular fractionation experiments in HepG2-cells and primary mouse hepatocytes (PMH) isolated from WT mice (*Ptpn6*^*f/f*^) and mice with liver-specific SHP-1 KO (*Ptpn6*^*H-KO*^) previously generated in our laboratory (3, 4). The analyses in both cell types showed that SHP-1 is localized in the cytoplasmic, membrane and nuclear fractions (Fig. 3, *B* and *C*). Similar to the transcription factor cAMP response element binding protein, SHP-1 was not only found in the soluble nuclear fraction, but also in the chromatin-bound nuclear fraction, where RNA pol II represented by its subunit POLR2C was localized (Fig. 3, *B* and *C*). Given the interaction between SHP-1 and POLR2J described above, we used a proximity ligation assay (PLA) to pinpoint the association of SHP-1 and RNA pol II (Fig. 3*D*). Forty-four percent of nuclei of SHP-1 WT cells as compared to only 5% of nuclei of SHP-1 KO cells used as negative control showed dots in the nucleus indicating that SHP-1 and the largest subunit POLR2A/RPB1, as a representative of RNA pol II, closely colocalized. Altogether, these experiments demonstrate that SHP-1 is recruited to chromatin, where transcriptional regulation occurs, and that SHP-1 endogenously interacts with the RNA pol II complex in nuclei.

### SHP-1 regulates *PCK1* transcript levels by controlling recruitment of RNA pol II to the *PCK1* promoter

**Figure 4:**
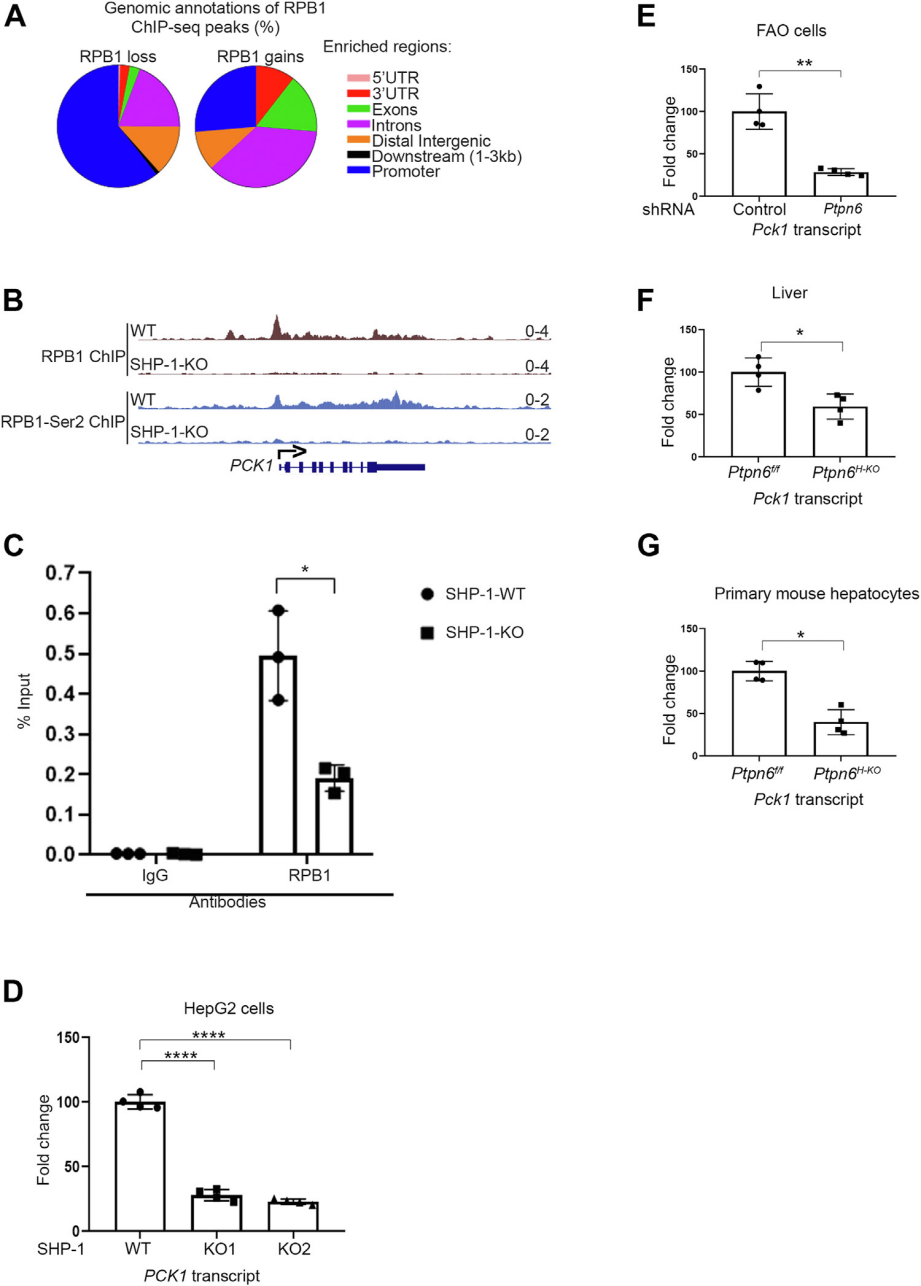
**Genome wide mapping of RPB1 binding regions in liver cells reveals the master regulator of gluconeogenesis *PCK1* as a target for SHP-1-mediated transcription regulation.***A*, distribution of significantly different RPB1 signal densities classified by human genomic annotations (hg38) in SHP-1 KO compared to SHP-1 WT cells. *B*, visualization of ChIP-seq data in the UCSC genome browser. ChIP-seq distribution for RPB1 and phospho-RPB1-Ser2 at *PCK1* gene. *C*, ChIP-qPCR validation of RPB1 binding on *PCK1* promoter (n = 3) (∗*p* < 0.05). *D*, expression levels of *PCK1* transcripts in SHP1 WT and KO HepG2 cells analyzed by qPCR. ∗∗∗∗*p* <0.0001 (n = 4). *E*, *Pck1*-mRNA levels in FAO cells with (*Ptpn6* shRNA) or without (control shRNA) knockdown of *SHP-1* determined by qPCR. ∗∗*p* < 0.01 (n = 4). *F*, levels of *Pck1* transcripts in liver lysates of *Ptpn6*^f/f^ and *Ptpn6*^H-KO^ mice analyzed by qPCR. ∗*p* < 0.05 (n = 4). *G*, levels of *Pck1* transcripts in primary hepatocytes isolated from *Ptpn6*^f/f^ and *Ptpn6*^H-KO^ mice analyzed by qPCR. ∗*p* < 0.05 (n = 4). ChIP, chromatin immunoprecipitation; PCK1, phosphoenolpyruvate carboxykinase 1; PTP, protein-tyrosine phosphatase; qPCR, quantitative PCR.

The presence of SHP-1 at the chromatin and its physical interaction with RNA pol II infer its direct role in transcriptional regulation. To investigate the functional relationship between SHP-1 and the transcription machinery, we profiled the genome-wide occupancy of POLR2A/RPB1, the largest subunit of the RNA pol II, in SHP-1 WT and SHP-1 KO HepG2 cells using chromatin immunoprecipitation coupled with massively parallel DNA sequencing (ChIP-seq) (30). As expected, we found a similar number of regions occupied by RPB1 in WT (46,045) and SHP-1 KO (41,605) cells. The distribution of RNA pol II-occupied regions was also similar between the WT and SHP-1 KO cells, with an average of 46% peaks at the promoter (44.6% and 47.4%, respectively), 40% in the gene body (40.4% and 39.4%) and 14.1% at distal regions (15% and 13.2%). To determine the functional consequences on RNA pol II recruitment, differential signal densities between WT and SHP1-KO cells were quantified. A total of 417 genomic regions harbored significant changes in RPB1 signal densities (*p* < 0.001) in SHP-1 KO *versus* SHP-1 WT cells (Fig. 4*A* and Table S2) with a striking bias for RPB1 losses (398 regions) compared to RPB1 gains (19 regions). The majority (60.5%) of RPB1 losses were located at gene promoters, while 25.1% were in the gene body (exon, intron, 5′ UTR, and 3′ UTR) and 14.3% at distal intergenic regions (Fig. 4*A*). By comparison, the small number of regions gaining RPB1 occupancy were found mostly in the gene body (63.1%). These results suggest that SHP-1 plays a role in the recruitment of RNA pol II at the promoter region at a subset of genes.

Among the genes that were decreased in RNA pol II density at their promoter in SHP-1 KO cells, we found *PCK1*, one of the master regulators of glucose homeostasis, which controls the second step of gluconeogenesis (18, 19). Since our previous *in vivo* findings showed reduced levels of fasting glucose and a decrease in hepatic gluconeogenesis in mice carrying a liver-specific SHP1-KO as compared to their WT counterparts (3, 4, 5), we focused our analysis on *PCK1* to expand on the role of SHP-1 in transcription and to further mechanistically understand the impact of SHP-1 on glucose metabolism. RPB1 ChIP-quantitative PCR (qPCR) analysis confirmed the decrease of RNA pol II occupancy at the *PCK1* promoter of SHP-1 KO compared to WT cells observed in the RPB1 ChIP-seq (Fig. 4, *B* and *C*). Observing a decrease in RNA pol II occupancy at the promoter region is not always associated with a loss in transcriptional activity. To ascertain that SHP-1 was modulating the transcriptional activity at the *PCK1* gene, we measured the levels of phosphorylation of RPB1 on serine 2, which is associated with transcriptional elongation (31). Density profiles of phospho-Ser2 RPB1 ChIP-seq showed decreased levels at the *PCK1* gene in SHP-1 KO cells (Fig. 4*B*). Together, these data imply that SHP-1 is involved in the transcriptional regulation of *PCK1*.

To corroborate our ChIP-data and determine whether SHP-1 generally controls the transcription of *PCK1* in hepatic cells, we measured mRNA levels in various liver cell lines and hepatic tissue by qPCR. We found a significant decrease of *PCK1* transcript levels in two independent HepG2 SHP-1 CRISPR KO cell lines (Fig. 4*D*). To validate the effects of SHP-1 on *PCK1* transcription in another liver cell line, we used *Ptpn6* shRNA to knockdown *Ptpn6* by about 50% in FAO rat hepatoma cells as compared to the control shRNA (Fig. S5*A*). As observed in HepG2 cells, we found a significant decrease in the *Pck1* transcript levels in FAO cells expressing *Ptpn6* shRNA (Fig. 4*E*). To further establish the *in vivo* relevance of our data, we determined the levels of *Pck1* transcripts in both liver tissues as well as PMH isolated from *Ptpn6*^*f/f*^ and *Ptpn6*^*H-KO*^ mice (4). We observed a significant decrease in *Pck1* transcript levels in both liver tissue (Fig. 4*F*) and isolated hepatocytes (Fig. 4*G*) from mice with a liver-specific ablation of SHP-1. The transcription of *G6Pc*, which encodes glucose-6-phosphatase (G6Pase), another central regulator of gluconeogenesis, is normally coregulated with *PCK1* (32, 33). Similar to *PCK1*, we found a significant decrease of *G6Pc* transcripts in the above described HepG2 SHP-1 KO cells (Fig. S6*A*) as well as in the FAO SHP-1 knockdown (KD) cells (Fig. S6*B*). Furthermore, primary hepatocytes and liver tissue from mice carrying a hepatocyte-specific SHP-1 KO showed a significant and nonsignificant reduction in *G6pc* transcript levels, respectively (Fig. S6, *C* and *D*). Taken together our data indicate that hepatic SHP-1 positively modulates *PCK1* and *G6Pc* transcript levels *in vitro* and *in vivo* further substantiating an important role for SHP-1 in transcription in this metabolic organ.

### STAT5-dependent binding of SHP-1 to the *PCK1* promoter regulates RNA pol II recruitment and PCK1 transcription

**Figure 5:**
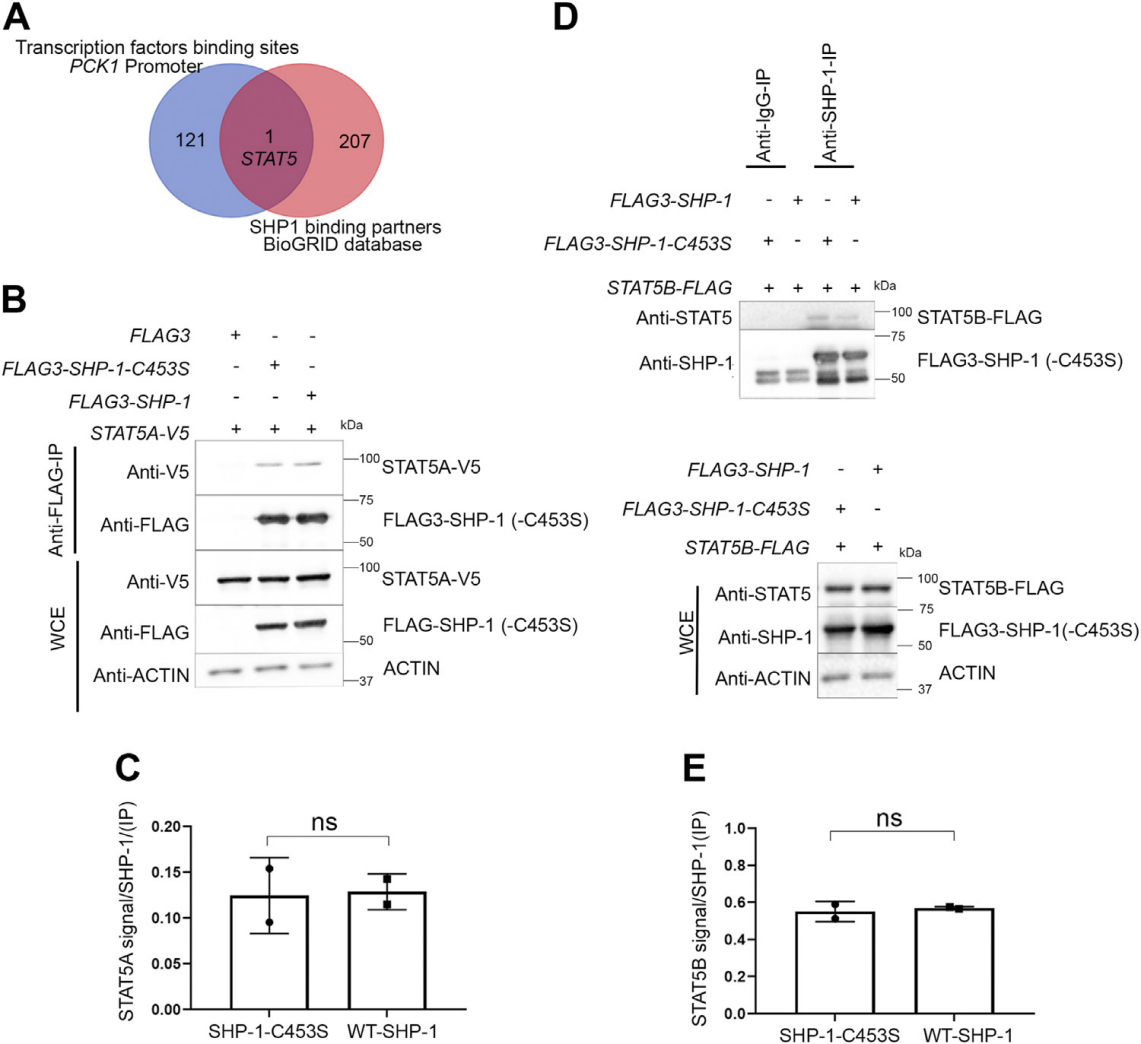
**SHP-1 interacts with STAT5 *in vitro* and shares a common binding region at the *PCK1*-promoter.***A*, Venn diagram predicting common factors by comparing transcription factors that bind to *PCK1*-promoter (PROMO) and proteins that interact with SHP-1 (BioGRID). *B*, Western blot analysis of co-immunoprecipitation showing interaction of SHP-1 and STAT5A in HepG2 cells using coexpression of V5-tagged *STAT5A* and FLAG-tagged *SHP-1*. WCE, whole cell extract (n = 2). *C*, quantification of SHP-1/STAT5A binding determined by densitometry using ImageJ (n = 2); ns = nonsignificant. *D*, Western blot analysis of co-immunoprecipitation showing interaction of SHP-1 and STAT5B in HepG2 cells using coexpression of FLAG-tagged *STAT5B* and FLAG-tagged *SHP-1*. Immunoprecipitation was performed using a SHP-1-specific antibody. WCE, whole cell extract (n = 2). *E*, quantification of SHP-1/STAT5B binding determined by densitometry using ImageJ (n = 2); ns = non-significant; BioGRID, Biological General Repository for Interaction Datasets; PCK1, phosphoenolpyruvate carboxykinase 1; STAT5, signal transducer and activator of transcription 5.

Since SHP-1 does not harbor any known DNA- or chromatin-binding domain we postulated that its recruitment to chromatin is indirect and requires the association with a transcription factor. We searched for transcription factor binding motifs in the *PCK1* promoter using the PROMO tool with a dissimilarity rate cutoff set to <15% (34) and compared the resulting list of transcription factors to the SHP-1-interacting partners listed in Biological General Repository for Interaction Datasets (BioGRID) (Table S7) (35). As seen in the Venn diagram in Figure 5*A*, the only common protein between the two datasets was the transcription factor STAT5, which refers to two nearly identical proteins, STAT5A and STAT5B. Interestingly, STAT5 was already implicated in the control of *PCK1* transcription in mammary gland epithelial cells and adipocytes (36, 37), but a potential role for SHP-1 in this mechanism has never been reported. Using co-IP experiments, we confirmed the association between STAT5A and STAT5B with SHP-1 (Fig. 5, *B* and *D*). An enzyme-substrate-interaction between SHP-1 and STAT5 was ruled out because STAT5A and STAT5B bound with the same affinity to WT SHP-1 and the substrate-trapping SHP-1-C453S mutant (Fig. 5, *C* and *E*). To further corroborate these findings, we analyzed the phosphorylation of STAT5 on tyrosine residue 694 (Y694), which is a readout for STAT5-activation. While STAT5-Y694 phosphorylation was not detected under basal conditions in serum-deprived cells of both genotypes, treatment of HepG2-and FAO-cells with growth hormone, a well-known activator of STAT5, induced STAT5-Y694 phosphorylation (Fig. S7, *A* and *B*), but depletion of SHP-1 did not further augment this effect. These results show that STAT5-phosphorylation does not change in a SHP-1-dependent manner and further confirm that STAT5 is not a substrate of SHP-1.

**Figure 6:**
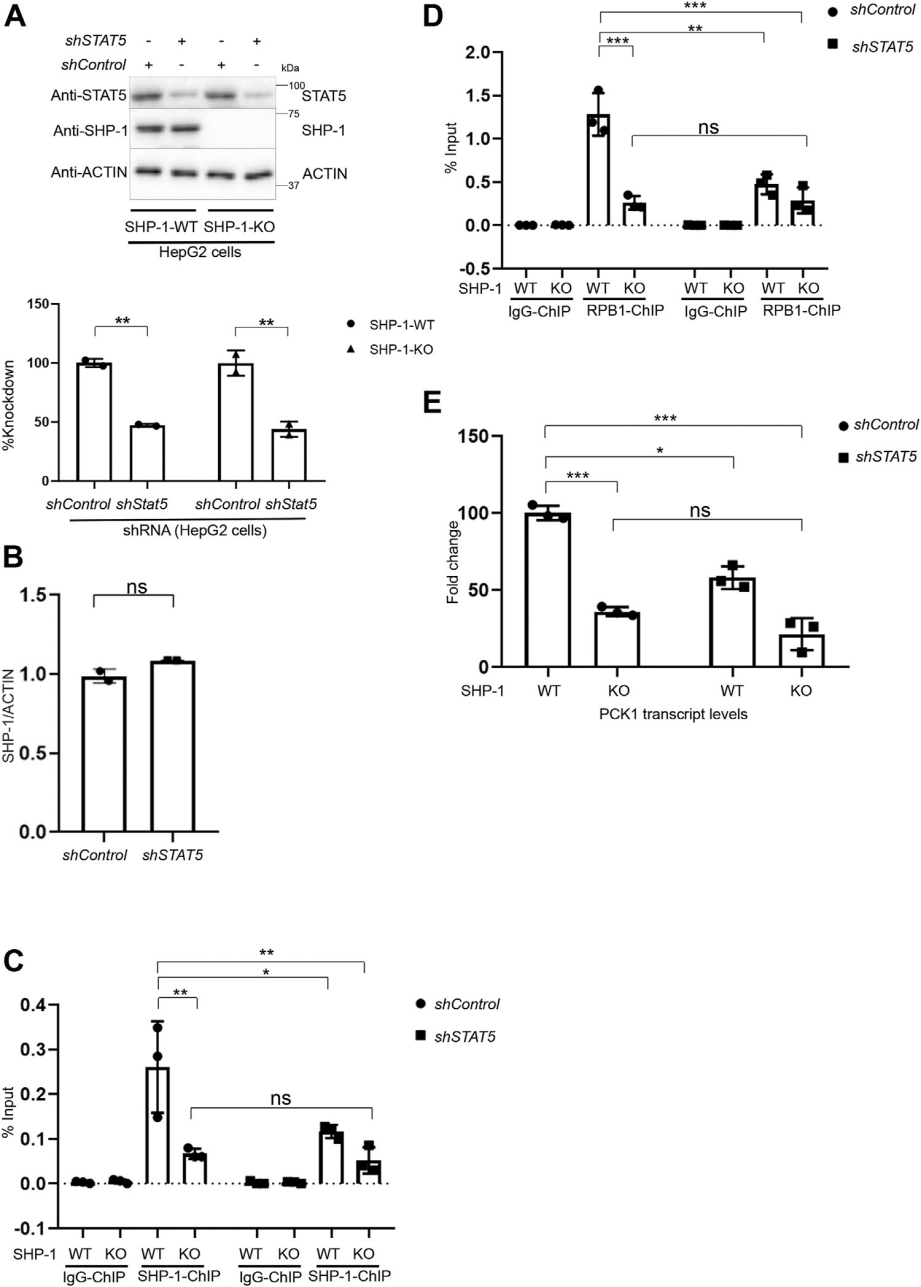
**STAT5-dependent recruitment of SHP-1 is required for the enrichment of RPB1 to the *PCK1* promoter thereby regulating *PCK1* transcription.***A*, confirmation by Western blot of STAT5 knockdown in HepG2 cells (SHP-1-WT or SHP-1-KO) using lentiviral infection with constructs carrying luciferase-specific (control) or *STAT5*-specific shRNA. Quantification of STAT5 knockdown levels determined by densitometry using ImageJ. ∗∗*p* < 0.01 (n = 2). *B*, quantification of SHP-1 levels in HepG2 WT-cells with (*shSTAT5*) or without (shControl) knockdown of STAT5 carrying either control shRNA or STAT5-specific shRNA determined by densitometry using ImageJ; ns = non-significant (n = 2). *C* and *D*, SHP-1 (*C*) and RPB1 (*D*) ChIP-qPCR at the *PCK1* promoter (n = 3). ∗*p* < 0.05, ∗∗*p* < 0.01, ∗∗∗*p* < 0.001. *E*, *PCK1*-mRNA levels in HepG2 cells with (*STAT5* shRNA) or without (Luc shRNA) knockdown of *STAT5* determined by qPCR (n = 3). ∗*p* < 0.05, ∗∗∗*p* < 0.001, ns: nonsignificant; ChIP, chromatin immunoprecipitation; PCK1, phosphoenolpyruvate carboxykinase 1; qPCR, quantitative PCR; STAT5, signal transducer and activator of transcription 5.

We next asked whether STAT5 recruits SHP-1 to the *PCK1* promoter, and how the SHP-1-STAT5 interaction affects the recruitment of RNA Pol II to the *PCK1* promoter. To answer these questions, we knocked down STAT5 in HepG2-WT and HepG2-SHP-1-KO cells using shRNAs and achieved a 50% reduction in STAT5 protein levels (Fig. 6*A*). Loss of STAT5 in HepG2-WT cells did not affect the total amount of SHP-1 (Fig. 6, *A* and *B*) ruling out that STAT5-mediated effects on SHP-1 are dependent on modulating its expression. We performed SHP-1 and RPB1 ChIP-qPCR analyses targeting the *PCK1* promoter in HepG2 SHP-1-WT and SHP-1-KO cells either expressing control or *STAT5* shRNA. Confirming the previously detected chromatin recruitment (Fig. 3), SHP-1 bound to the *PCK1* promoter in SHP-1-WT cells (Fig. 6*C*). STAT5 KD significantly reduced the recruitment of SHP-1 indicating that STAT5 is required for the binding of SHP-1 to the *PCK1* promoter. Moreover, we found that the enrichment of RPB1 on the *PCK1* promoter was significantly decreased after KD of STAT5 as well as in SHP-1-KO cells, but KD of STAT5 in SHP-1 KO cells did not further impact this reduction (Fig. 6*D*). Reduced levels of RNA pol II at the *PCK1* promoter correlated with significantly decreased *PCK1* transcript levels in STAT5 KD, SHP-1 KO, and STAT5 KD/SHP-1 KO cells (Fig. 6*E*). Taken together, these results suggest that SHP-1 recruitment to the promoter of *PCK1* requires STAT5 for RNA pol II activation.

### Gluconeogenesis is controlled by SHP-1 together with STAT5

**Figure 7:**
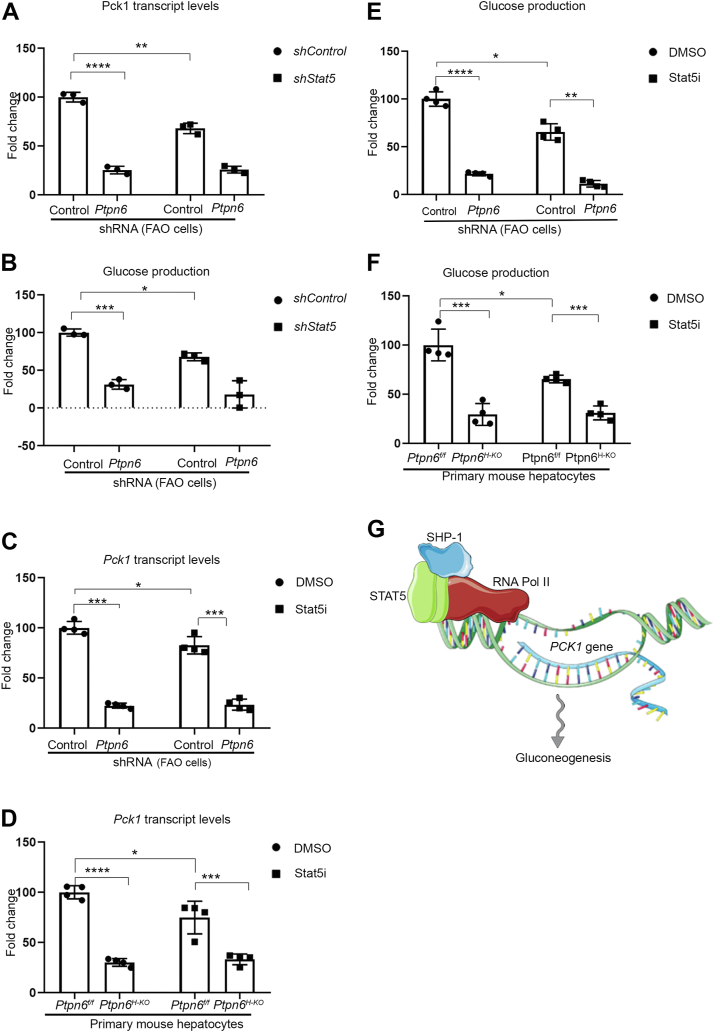
**Gluconeogenesis is controlled by SHP-1 *via* STAT5.***A*, *Pck1*-mRNA levels in SHP-1 WT and SHP-1-KD FAO cells with or without STAT5-specific shRNA determined by qPCR (n = 3). ∗∗∗∗ *p* < 0.0001, ∗∗*p* < 0.01. *B*, determination of hepatic glucose production in SHP-1 WT and SHP-1-KD FAO cells with or without STAT5-specific shRNA (n = 3). ∗∗∗ *p* < 0.001, ∗*p* < 0.05. *C*, *Pck1*-mRNA levels in FAO cells with (*Ptpn6* shRNA) or without (control shRNA) knockdown of *SHP-1* in response to DMSO or STAT5 inhibitor determined by qPCR (n = 4). ∗∗∗*p* < 0.001, ∗*p* < 0.05. *D*, *Pck1*-mRNA levels in PMH isolated from *Ptpn6*^f/f^ and *Ptpn6*^H-KO^ mice in response to DMSO or STAT5 inhibitor determined by qPCR (n = 4). ∗∗∗∗*p* < 0.0001, ∗∗∗*p* < 0.001, ∗*p* < 0.05. *E*, determination of hepatic glucose production in FAO cells with (*Ptpn6* shRNA) or without (control shRNA) *SHP-1* knockdown in response to DMSO or STAT5 inhibitor. (n = 4) ∗∗∗∗*p* < 0.0001, ∗∗*p* < 0.01, ∗*p* < 0.05. *F*, determination of hepatic glucose production in PMH isolated from Ptpn6^f/f^ and Ptpn6^H-KO^ in response to DMSO or STAT5 inhibitor (n = 4). ∗∗∗*p* < 0.001, ∗*p* < 0.05. *G*, model depicting transcriptional regulation of *PCK1* transcription mediated by SHP-1, STAT5, and RNA pol II resulting in control of gluconeogenesis. This figure was partly generated using Servier Medical Art (https://smart.servier.com), provided by Servier, licensed under a Creative Commons Attribution 3.0 unported license. DMSO, dimethyl sulfoxide; PCK1, phosphoenolpyruvate carboxykinase 1; PMH, primary mouse hepatocytes; PTP, protein-tyrosine phosphatase; qPCR, quantitative PCR; RNA pol II, RNA polymerase II; STAT5, signal transducer and activator of transcription 5.

PCK1 is a major regulator of gluconeogenesis. To investigate the importance of SHP-1 and STAT5 in a physiological context, we measured *PCK1* transcript levels and glucose production in FAO cells, a well-established model for glucose metabolism studies (38, 39, 40, 41). To better understand the relationship between SHP-1 and STAT5 in this process we used depletion of STAT5 in the SHP-1 WT or SHP-1 KD background and measured *Pck1* transcript levels and glucose production. KD of STAT5 using shRNA in FAO SHP-1-WT and FAO SHP-1-KD cells reduced the levels of STAT5 protein in these cells by 50% (Fig. S5*B*). We found that depletion of SHP-1 as well as STAT5 significantly decreased *Pck1* transcript levels as well as glucose production (Fig. 7, *A* and *B*). Importantly, STAT5 KD did not further decrease *Pck1* transcript levels and the glucose production capacity in cells depleted of SHP-1, suggesting that SHP-1 and STAT5 act on the same pathway in the regulation of gluconeogenesis mediated by control of *PCK1* transcription. Furthermore, these data confirm the results obtained in HepG2 cells (Fig. 6) and show that the SHP-1-STAT5-mediated modulation of RNA pol II recruitment to the *PCK1* promoter followed by differential *PCK1* transcription is mirrored by metabolic changes as reflected by hepatic glucose production.

To further validate our data obtained in the STAT5 depleted FAO cells, we used a pharmacological approach to inhibit STAT5 in FAO cells but also in freshly isolated mouse hepatocytes (PMH), the gold standard for *ex vivo* glucose production assays (42). We treated FAO SHP-1-WT and FAO SHP-1-KD cells, as well as PMH isolated from *Ptpn6*^*f/f*^ and *Ptpn6*^*H-KO*^ mice with a STAT5 inhibitor (CAS 285986-31-4) (43) and assessed *PCK1* transcription by qPCR and gluconeogenesis as a functional readout. We found in both hepatic cellular models that STAT5 inhibitor treatment (Stat5i) of SHP-1-WT cells significantly reduced *Pck1* transcript levels, but not to the same extent as observed in SHP-1 KD cells exhibiting no further decrease in *Pck1* transcript levels indicating that SHP-1 and STAT5 act on the same pathway (Fig. 7, *C* and *D*). Matching the *Pck1* transcript level data, we observed a significant decrease of glucose production in FAO-cells and PMH after treatment of SHP-1 WT cells with STAT5 inhibitor. A larger decrease in SHP-1 KD cells, which was not significantly changed after STAT5 inhibitor treatment of these cells, was noticed (Fig. 7, *E* and *F*).

## Discussion

We discovered a new metabolic function for SHP-1 in the liver, whereby the PTPase activates hepatic gluconeogenesis through coactivation of *PCK1*-transcription in liver cells. Moreover, we elucidated the molecular mechanism of this transcriptional regulation by demonstrating that the STAT5-dependent recruitment of SHP-1 controls RNA pol II at the *PCK1* promoter.

SHP-1 is a major regulator of cytokine and immune receptor signaling in hematopoietic cells, and we have shown that it negatively modulates insulin signaling in metabolic tissues (1, 4, 13). In the present study, we have identified a novel, noncanonical SHP-1 function as a key promoter of hepatic gluconeogenesis. Despite being mainly characterized as a cytoplasmic protein-tyrosine phosphatase, SHP-1 is also found in nuclei of epithelial cells (8, 9, 44). Here, we report for the first time that SHP-1 is associated with the promoter region of the *PCK1* gene. Furthermore, we provide strong evidence that SHP-1 interacts with RNA pol II and the transcription factor STAT5. The recruitment of SHP-1 to the *PCK1* promoter was dependent on STAT5. Depletion of SHP-1 not only reduced recruitment of RNA pol II to the *PCK1* promoter, but also decreased *PCK1* transcript levels and subsequently gluconeogenesis. STAT5 downregulation or inhibition had similar effects on RNA pol II recruitment, *Pck1* transcription and glucose production. Thus, we propose a conceptual model whereby STAT5-bound SHP-1 regulates *Pck1* transcription through RNA polymerase II recruitment to the *Pck1* promoter, thereby modulating glucose production by liver cells (Fig. 7*G*).

These findings provide a key mechanism to explain our previous observations that mice with hepatocyte-specific deletion of SHP-1 (*Ptpn6*^*H-KO*^) exhibited lower fasting glycemia as compared to their littermate WT controls (3, 4). These mice also exhibited a markedly reduced hepatic glucose production rate (3). The pathophysiological relevance of these findings is further demonstrated by the observation that SHP-1 expression is increased in the liver of high-fat fed obese mice and that SHP-1 genetic deletion reduced fasting hyperglycemia but also fully blunted the elevated glucose production in these obese mice (3).

Glucagon (45) cortisol (46), epinephrine (47), and growth hormone (33) are some upstream signals for gluconeogenesis. These signals collectively increase the expression and activity of enzymes involved in gluconeogenesis, leading to an enhanced production of glucose from noncarbohydrate sources. In the context of gluconeogenesis, the relationship between SHP-1 and the upstream signals mentioned earlier is not direct or well-established. However, it is worth noting that SHP-1 can be part of complex signaling networks that intersect with the pathways regulating gluconeogenesis. For example, SHP-1 has been implicated in the modulation of insulin signaling and insulin resistance (3, 4, 13). Disruption of insulin signaling can dysregulate glucose homeostasis and potentially affect gluconeogenesis. The specific relationship between SHP-1 and the upstream signals for gluconeogenesis will require further investigations.

SHP-1 has been relatively underappreciated in terms of its metabolic functions. Its role as a phosphatase is crucial in regulating cellular signaling processes. However, despite its significance, there is a knowledge gap regarding the identification of novel SHP-1 substrates (48) involved in gluconeogenesis. This exploration will provide valuable insights into the intricate SHP-1 phosphatase-dependent molecular mechanisms underlying gluconeogenesis.

Coactivators are a diverse group of proteins which modulate transcription in a variety of different ways (49, 50). These include modification of chromatin or unraveling DNA through enzymatic action of the coactivators and functioning as an adapter between the transcription factor and RNA pol II to direct recruitment of the transcriptional apparatus. We found that the tyrosine-phosphatase SHP-1 shows the characteristics of a typical transcriptional coactivator. SHP-1 binds to the transcription factor STAT5, which is required for the association of SHP-1 to chromatin. SHP-1 interacts with RNA pol II and its invalidation reduces recruitment of RNA pol II to the regulatory regions of the *PCK1* gene and *PCK1* transcription. Despite using four different domain prediction tools namely Motif Scan (MyHits, SIB), InterPro 5, MOTIF (GenomeNet) and CD-Search (Conserved Domain Databases), we were not able to identify a DNA- or chromatin-binding domain in SHP-1 corroborating that the recruitment of SHP-1 to the *PCK1* promoter is mediated by the interaction with promoter-bound STAT5. However, the exact spatial and temporal formation of the SHP-1-STAT5 complex and its association with chromatin have yet to be determined. Since a plethora of transcription factors including cAMP responsive element binding protein (51) and Forkhead box protein O1 (FOXO1) (22) have been described for control of *PCK1* transcription, it will be interesting to elucidate whether any other transcription factors are involved in the recruitment of SHP-1 to the *PCK1* promoter and how the SHP-1-STAT5 complex is integrated into this circuitry of transcriptional regulation.

Recently, phosphatase and tensin homologue deleted on chromosome ten (PTEN), a dual specificity phosphatase (52), as well as the insulin receptor (53) were shown to regulate gene transcription in a very similar fashion to SHP-1 by interacting directly with the transcription machinery. These studies did not provide conclusive, mechanistic evidence for the requirement of PTEN phosphatase activity, nor the insulin receptor kinase activity for direct transcriptional regulation. The increased interaction between POLR2J and the substrate-trapping SHP-1-C453S-mutant as compared with the WT form of SHP-1 suggests that POLR2J might be a substrate for SHP-1. In addition, while the preferential binding of POLR2J to the SH2-domains of SHP-1 would generally imply a tyrosine phosphorylation-dependent process, we were not able to detect any tyrosine phosphorylation of POLR2J using several complementary molecular approaches. Therefore, the interaction between SHP-1 and POLR2J is not an enzyme-substrate interaction and tyrosine phosphorylation is not involved. Identification of the binding motif in POLR2J remains to be elucidated. SHP-1 has been implicated in the modulation of transcriptional processes by dephosphorylating different transcription factors (11, 12), but phosphatase activity-independent functions for SHP-1 have been demonstrated for other cellular processes such as leukocyte-associated immunoglobulin-like receptor 1 and cytokine signaling (54, 55). It will be interesting to study whether SHP-1 phosphatase activity is required for the regulation of *PCK1* transcription by dephosphorylating, for example, any other nearby transcription factors.

In recent years, evidence mounted that STAT5 is involved in the regulation of *PCK1* transcription and gluconeogenesis, but the mechanistic details are not fully elucidated and data are controversial. While it was demonstrated that elevated levels of suppressor of cytokine signaling-2 cause reduced *PCK1* expression and glucose production by interfering with STAT5 activation (56), growth hormone was reported to induce *PCK1* transcription through tyrosine-phosphorylation of STAT5 (32). In contrast, it was shown that lack of STAT5 activation through abrogation of growth hormone signaling or even increased STAT5-tyrosine-phosphorylation did not result in an alteration of *PCK1* expression (57, 58). Phosphorylation of STAT proteins at a C-terminal tyrosine residue generally leads to transcriptional activation by enhancing dimerization, nuclear localization, and binding of STATs to DNA (59, 60). While some studies reported that SHP-1 can dephosphorylate STAT5 *in vitro* (61, 62), another study suggests that STAT5 is not a substrate of SHP-1, but rather of SHP-2 (63). Our experiments revealed that STAT5 together with SHP-1 plays an important role in *PCK1* transcriptional regulation and gluconeogenesis, but tyrosine-phosphorylation does not seem to be required for STAT5-SHP-1 interaction or for this STAT5-SHP-1-mediated process. STAT5 bound equally well to WT-SHP-1 and a substrate-trapping SHP-1 mutant, and STAT5 tyrosine-phosphorylation was not increased in SHP-1 KO cells compared to SHP-1 WT cells inferring that their interaction is not dependent on tyrosine-phosphorylation and that STAT5 is not a direct substrate for SHP-1. Our data support tyrosine-phosphorylation-independent mechanisms in this process adding to the growing list of evidence that unphosphorylated STATs (uSTAT) can play important roles in transcriptional regulation (64). Indeed, uSTAT3 has been reported to form a complex with unphosphorylated NFκB to translocate into the nucleus and activate a subset of NFκB-dependent genes (65). Additionally, uSTAT6 cooperates with the transcriptional coactivator p300 to enhance COX-2 expression (66). Furthermore, uSTAT5 by regulating heterochromatin stability through interaction with HP1α (67) or in cooperation with the transcription factor CTCF, regulates different transcriptional programs (68).

Our ChIP-analysis revealed that SHP-1 might be involved in the transcriptional modulation of many other genes than *PCK1* that may also carry key functional roles in the modulation of glucose metabolism in liver and other metabolic tissues. Future work beyond the scope of this study will consist of expanding our analysis to determine whether the mechanism by which SHP-1 exerts transcriptional regulation at different genes. Furthermore, other factors may be involved in the specific interactions between SHP-1, STAT5, and RNA pol II. The interplay of these factors fine-tuning SHP-1 recruitment to chromatin will need to be further investigated.

In summary, we report the novel observation that SHP-1 directly associates with the *PCK1* promoter through STAT5 and RNA pol II and that its presence is required for *PCK1* expression, thereby promoting hepatic gluconeogenesis. Our data suggest that SHP-1 nuclear localization is necessary to control hepatic gluconeogenesis through directly regulating STAT5-mediated *PCK1* transcription, thus adding a novel nuclear mechanism to the repertoire of SHP-1 metabolic functions.

## Experimental procedures

### Cell lines and treatment

All cell lines were cultured at 37 °C in a humidified atmosphere containing 5% CO2. HepG2 cells were cultured in Dulbecco’s modified Eagle’s medium (DMEM), low glucose supplemented with 10% fetal bovine serum (FBS). Flp-In 293 T-REx cell lines were cultured in DMEM (high glucose) containing 10% FBS. The rat hepatoma FAO cells were cultured in RPMI supplemented with 10% FBS. PMH were isolated from WT mice (*Ptpn6*^*f/f*^) and mice with liver-specific SHP-1 KO (*Ptpn6*^*H-KO*^) previously used in our laboratory (3, 4). HepG2 cells and FAO cells were serum starved overnight and then treated with human growth hormone (500 ng/ml) and rat growth hormone (500 ng/ml), respectively, for up to 60 min.

### Mammalian cell transfection

HepG2 and Flp-In 293 T-REx cell lines were transfected using jetPRIME (Polyplus-transfection) and PureFection (System Biosciences) transfection reagents according to the manufacturer’s instructions with cell confluence between 60 and 80%.

### DNA constructs

To generate inducible *FLAG3-SHP-1* constructs for expression in Flp-In 293 T-REx cells, *SHP-1*- and *SHP-1-C453S*-complementary DNAs (cDNAs) were PCR-amplified from existing vectors in our laboratory and cloned using *Hind*III and *Eco*RI restriction sites into pcDNA5/FRT/TO containing a sequence with three consecutive FLAG-epitopes for N-terminal tagging. *SHP-1*-fragments (*SH2-SH2* and *PTPase*) were PCR-amplified from the pcDNA5/FRT/TO-FLAG3-SHP-1 construct and cloned using *Hind*III and *Eco*RI to generate pcDNA5/FRT/TO-FLAG3-SH2-SH2 and pcDNA5/FRT/TO-FLAG3-PTPase constructs. *SHP-1*-substitution mutants (D419A, R459M and A455T) and *SHP-1*-deletion mutant (ΔP=Δ451–475) were created by Q5 site-directed mutagenesis (New England Biolabs) and QuikChange site-directed mutagenesis (Agilent), respectively, using pcDNA5/FRT/TO-FLAG3-SHP-1 as template. These constructs were also used for constitutive overexpression in HepG2-cells. To create *Myc3-POLR2J*- and *Myc3-POLR2C*-constructs, *POLR2J*- and *POLR2C*-cDNAs (vectors containing Human MGC Verified FL cDNA from OpenBiosystems/Thermo Fisher Scientific) were PCR amplified and cloned using *Bam*HI and *Xba*I restriction sites into pcDNA3.1-Myc3 (a kind gift from Dr Sabine Elowe). The pLX304-STAT5A-V5 plasmid was a kind gift of Dr Mathieu Laplante. The pAD-CMV-STAT5B-CMV-GFP plasmid was purchased from Addgene (#83257). To create a *Myc3-STAT5A*-construct, *STAT5A*-cDNA was PCR-amplified from the pLX304-STAT5A-V5 plasmid and cloned as a *Bgl*II-*Xho*I-fragment into pcDNA3.1-Myc3 cut with *Bam*HI and *Xho*I.

### Generation of stable Flp-In T-Rex 293 cells inducibly expressing SHP-1 constructs

Flp-In 293 T-REx cell lines expressing either *3xFLAG*, *FLAG3-SHP-1* (WT), or *FLAG3-SHP-1-C453S* (DN) were generated using Flp-In T-REx 293 cell line system as described by the manufacturer (Invitrogen). Briefly, Flp-In T-REx-293 cell cells were cotransfected with pcDNA5/FRT/TO expression vectors containing either *3xFLAG*, *FLAG3-SHP-1*, or *FLAG3-SHP-1-C453S* together with Flp recombinase vector pOG44. Transfected cells were selected using Hygromycin B (200 μg/ml), and expression was confirmed by immunoblotting.

### Co-IP, tyrosine-phosphorylation, immunoblotting, and mass spectrometry

Flp-In T-REx 293 cells stably expressing either 3xFLAG, FLAG3-SHP-1, or FLAG3-SHP-1-C453S for affinity-purification mass spectrometry (AP-MS) were treated or not with 1 μM insulin for 30 min after serum-deprivation for 39 h. To express inducible constructs, cells were treated with tetracycline (1 μg/ml) for 27 h (AP-MS) or 24 h (co-IP, tyrosine-phosphorylation) before harvesting. For co-IP, HepG2 cells were cotransfected with either FLAG3-SHP-1, FLAG3-SHP-1-C453S or empty 3xFLAG vector and 3xMyc-tagged POLR2C, 3xMyc-tagged POLR2J, V5-tagged STAT5A or FLAG-tagged STAT5B and harvested after 24 h. To look at tyrosine-phosphorylation, Flp-In T-REx 293 cells stably expressing either 3xFLAG or FLAG3-SHP-1-C453S were transfected with 3xMyc vector or Myc3-POLR2J and expression was induced as described above. Twenty-four hours later, cells were treated with 20 μM bpV(HOpic) or left untreated and then harvested. HepG2 cells were cotransfected with 3xFLAG- or FLAG3-SHP-1-C453S- and 3xMyc- or Myc3-POLR2J-constructs, or singly transfected with 3xMyc-, Myc3-POLR2J, or Myc3-STAT5A-constructs. Twenty-four hours after transfection, cells were treated for 30 min with 20 μM bpV(HOpic) or left untreated and then harvested. For co-IP/IP, cells were lysed in lysis buffer containing 20 mM Tris–HCl pH 7.5, 140 mM NaCl, 1 mM of CaCl_2_ and MgCl_2_, 10 mM NaF, 1% NP-40, 10% glycerol, 2 mM Na-Vanadate, 1 mM PMSF and protease inhibitors from Roche. Lysates were centrifuged and protein was quantified using the bicinchoninic acid assay method. One milligram of total protein was incubated for 2 h with anti-FLAG M2 affinity gel, anti-FLAG M2 magnetic beads, anti-c-Myc agarose affinity gel (Sigma-Aldrich) or Protein G Dynabeads preincubated with isotype control mouse IgG2b or anti SHP-1 antibody, followed by five washes with buffer containing 1× PBS, 2 mM Na-Vanadate, and protease inhibitor. Proteins were eluted using 1× Laemmli sample buffer. The expression of proteins was determined by immunoblotting, which was done as described before (4, 13), using specific antibodies listed in Table S6.

For AP-MS, samples were prepared as reported before (25) and analyzed by liquid chromatography-tandem mass spectrometry on a Thermo LTQ instrument. The *Homo sapiens* RefSeq V29 appended with the reversed decoy sequences was used for searching with the Mascot search engine allowing for methionine oxidation as a variable parameter. Evaluation of the recovered proteins in the SHP-1 purifications against the nine selected negative controls was performed using SAINTexpress (28). Briefly: with each of the two baits (SHP-1 WT and SHP-1 substrate trapping mutant), for each prey (protein detected in a pull-down), the two highest spectral counts across four analyses (from duplicate purifications of samples treated or not with insulin) were selected for SAINTexpress scoring with default options. The SAINTexpress file was downloaded and used as an input for the visualization tool ProHits-viz; high-confidence interactors were those deemed by SAINTexpress to fall within 1% of the Bayesian False Discovery Rate (69).

### Generation of single-cell cultures of SHP-1 KO HepG2 cells

The single guide RNAs (sgRNAs) targeting Shp1 were designed using CRISPR DESIGN (http://crispr.mit.edu/) (70). The sgRNA with a high score and low off-target score were selected. Guide RNAs targeting different exons of PTPase domains and nontargeting guides were synthesized, annealed, and cloned in px459 V2.0 vector as described by Ran *et al.*, 2013 (70) (Table S3). The sgRNA constructs were sequenced using U6 primer. HepG2 cells were seeded in a six-well plate (0.5 million per well) and were either left untransfected or transfected with Sg4, Sg1, and Sg6 (nontargeting guide) constructs. Puromycin was added (2 μg/ml) after 24 h of transfection for a period of 3 days. After 3 days, cells were counted, and 500 cells were seeded in a 150 mm dish and incubated for 7 to 10 days at 37 °C with 5% CO_2_. Single-cell clones were transferred into 24-well plates using the filter disc method. When single-cell cultures were sufficiently grown, protein lysates were prepared and the expression of SHP-1 was determined by immunoblotting (Fig. S4).

### Cell fractionation

HepG2-cells expressing WT SHP-1 or CRISPR-KO-SHP-1 cell fractionations were performed using a subcellular protein fractionation kit for cultured cells (Thermo Fisher Scientific) as per the manufacturer’s instructions. Enrichment of marker proteins and SHP-1 was determined by immunoblotting.

### Proximity ligation assay

HepG2 cells (WT or CRISPR-KO-SHP-1) were plated in complete DMEM low glucose media on poly-l-lysine coated coverslips. Cells were fixed in 4% paraformaldehyde for 20 min followed by quenching (50 mM NH_4_Cl) and blocking (PBS containing 10% donkey serum, 0.3% Triton X100) for 1 h at RT. Cells were incubated with primary antibodies (anti-SHP-1, 1:50, sc-287, Santa Cruz; anti-RPB, 1:20, ab817, Abcam) at 4 °C overnight followed by incubation with the PLA probes, ligase, and polymerase using the Duolink *In Situ* Orange Starter Kit Mouse/Rabbit protocol as provided by the manufacturer (Millipore Sigma). Images were acquired with the LSM800/AxioObserver.Z1 (Zeiss) using a 63× oil objective. Nuclear dots were evaluated in a total of at least 400 nuclei.

### ChIP-seq

HepG2 cells (WT or CRISPR-KO-SHP-1) were cultured in 150 mm dishes. Cells were washed three times with PBS and 5 × 10^7^ cells for each ChIP-seq fixed with 1% formaldehyde for 10 min. Cross-linked cells were quenched using glycine for 5 min at room temperature (RT). Cells were washed twice with ice-cold PBS and harvested using a silicon scraper. Cells were transferred to 50 ml falcon and centrifuged at 1350*g* for 5 min. Pellets obtained were washed again with ice-cold PBS. Cell pellets were suspended in LB1 buffer (50 mM Hepes-KOH, pH 7.5, 140 mM NaCl, 1 mM EDTA, 10% glycerol, 0.5% IGEPAL, 0.25% Triton X-100, PMSF and protease inhibitor cocktail) and incubated at 4 °C for 10 min with rotation. Pellets were obtained using centrifugation. The cell pellet was suspended in LB2 buffer (10 mM Tris–HCl, pH 8.0, 200 mM NaCl, 1 mM EDTA, and 0.5 mM EGTA supplemented with protease inhibitors) and incubated for 10 min at RT with rotation. Lysates were centrifuged at 1350*g* for 5 min and pellets were suspended in sonication buffer (20 mM Tris–HCl pH 8.0, 150 mM NaCl, 2 mM EDTA pH 8.0, 0.1% SDS, and 1% Triton X-100 supplemented with protease inhibitors). Lysates were sonicated using Bioruptor instrument (Diagenode) for a total of 6 min (high intensity, 12 cycles of 30 s ON and 30 s OFF) at 4 °C to obtain a chromatin fragmentation of 200 to 900 bp. Protein G dynabeads preblocked in blocking solution (0.5% bovine serum albumin in PBS) were incubated with antibodies (RPB1 and pSer2-RPB1) at 4 °C for 6 h. Protein G Dynabead-antibody complexes were washed three times with 1 ml blocking solution, then added to chromatin preparations and incubated overnight at 4 °C. Some portions of the chromatin were left untreated and used as whole-cell extract (WCE) control. Beads were washed once with Wash buffer B (20 mM Tris–HCl pH 8.0, 150 mM NaCl, 2 mM EDTA pH 8.0, 0.1% SDS, 1% Triton X-100, and protease inhibitors), followed by Wash buffer C (20 mM Tris–HCl pH 8.0, 500 mM NaCl, 2 mM EDTA pH 8.0, 0.1% SDS, 1% Triton X-100 and protease inhibitors), and Wash Buffer D (10 mM Tris–HCl pH8.0, 250 mM LiCl, 1 mM EDTA pH 8.0, 1% NP40, and protease inhibitor) and TE buffer (10 mM Tris pH 8.0, 1 mM EDTA, 50 mM NaCl and protease inhibitors). Complexes were eluted off the beads using elution buffer containing 50 mM Tris–HCl pH 8.0, 1 mM EDTA pH 8.0, and 50 mM NaCl. Beads were collected and incubated at 65 °C overnight to reverse cross-linking. In parallel, WCE were also reverse cross-linked. Samples were subsequently treated with RNase A (Ambion) at 37 °C for 2 h followed by Proteinase K (Invitrogen) treatment at 55 °C for 30 min. DNA was purified using the phenol–chloroform method. Libraries were constructed from ChIP- and WCE-samples using NEBNext Ultra II DNA library prep kit (New England Biolabs) according to the manufacturer’s instructions and single-end sequenced (50 bp) on an Illumina HiSeq 2500 instrument at the Next-Generation Sequencing Platform of the CHU de Québec-Université Laval Research Center (CRCHUQ).

### ChIP-seq data analysis

Analysis of raw sequencing reads was performed using the MUGQIC ChIP-Seq pipeline (71). Briefly, reads were trimmed for adapter sequences using Trimmomatic (72). High-quality reads were aligned to the human reference genome (GRCH38/hg38) with the Burrows–Wheeler aligner (73). Narrow peaks were called using MACS2 (74), using the corresponding input DNA as background. For visualization, reads were extended 200 bp using the bamCoverage function from deepTools (75). Gene tracks were created using the University of California, Santa Cruz (UCSC) Genome Browser (Table S7) (76). Differential binding between SHP-1-KO and WT was assessed using csaw (77) with corrected *p*-values using the Benjamini-Hochberg procedure.

### Micrococcal nuclease ChIP

Frozen cross-linked cell pellets of HepG2 cells (WT or CRISPR-KO-SHP-1) were thawed and suspended in LB1 buffer. Cell pellets were homogenized with 15 strokes of a Dounce glass pestle A and 30 strokes of pestle B on ice. Cell pellets were obtained upon centrifugation, suspended in LB2 buffer, and incubated for 10 min at RT with rotation. Lysates were centrifuged and suspended in MNase digestion buffer (New England Biolabs) containing 500 gel units of Micrococcal nuclease (Fig. S8*A*). Cell pellets were incubated at 37 °C for 20 min with shaking (300 rpm). The reaction was stopped by adding EDTA. Cell pellets were centrifuged and suspended in ChIP-SDS lysis buffer (1% SDS, 10 mM EDTA, and 50 mM Tris, pH 8.1). Pellets were homogenized with 100 strokes of Dounce pestle B on ice. Homogenized pellets were observed under the microscope (Fig. S8*B*). Pellets were subjected to three-freeze thaw cycles using liquid nitrogen. Lysates were diluted in ChIP dilution buffer (0.01% SDS, 1.1% Triton X-100, 1.2 mM EDTA, 16.7 mM Tris–HCl, pH 8.1, and 167 mM NaCl) and precleared with preblocked Protein G Dynabeads at 4 °C for 1 h. At this point, an aliquot of samples was taken for input controls. The precleared lysates were incubated with antibody (SHP-1 or RPB1)-pre-blocked protein G Dynabead complexes overnight at 4 °C. Beads were consecutively washed each time for 15 min at 4 °C with low salt immune complex wash buffer (0.1% SDS, 1% Triton X-100, 2 mM EDTA, 20 mM Tris–HCl, pH 8.1, 150 mM NaCl), followed by high Salt Immune Complex Wash Buffer (0.1% SDS, 1% Triton X-100, 2 mM EDTA, 20 mM Tris–HCl, pH 8.1, 500 mM NaCl), LiCl Immune Complex Wash Buffer (0.25 M LiCl, 1% IGEPAL-CA630, 1% deoxycholic acid (sodium salt), 1 mM EDTA, 10 mM Tris, pH 8.1) and finally with TE buffer (10 mM Tris–HCl, 1 mM EDTA, pH 8.0). A small portion of the beads was eluted using Laemmli buffer and the amount of immunoprecipitated SHP-1 was determined by immunoblotting (Fig. S8*C*). The rest of the complexes were eluted off the beads and reverse cross-linking, RNAse-, and Proteinase K-treatment was done as described earlier. Same treatments were given to the input controls. DNA was purified using Qiagen PCR purification kit as per the manufacturer’s instruction. ChIP was analyzed using qPCR. The sequences of the oligonucleotides used in the study are shown in Table S4. The value of enrichment was calculated relative to the input controls.

### Lentivirus preparation and generation of KO cell lines

Lentiviruses were prepared by transfecting the lentiviral constructs encoding *shControl* (a kind gift from M. Laplante), *shPtpn6* or *shStat5* (Table S5) along with psPAX2 and pMD2G into 293T cells using jetPRIME transfection reagent as per the manufacturer’s instruction. Viruses containing supernatants were collected 48 h after transfection and filtered using a 0.45 μm filter (78).

FAO or HepG2 cells were transduced with lentiviral supernatant (*shControl*, *shPtpn6*, or *shStat5*) in the presence of polybrene (8 μg/ml). After 24 h of infection cells were washed three times with media and maintained in fresh virus-free media for 24 h. Then, cells were split and selected using puromycin (2 μg/ml) for 3 days. KD efficiency of shRNAs was assessed by Western blotting.

### RNA isolation and RT-qPCR

Total RNA was isolated using the Zymo research kit as per the manufacturer’s instructions. The purity and quantity of RNA were assessed using BioDrop. Two micrograms of total RNA was reverse transcribed using high capacity cDNA reverse transcription kit (Applied biosystems) supplied with random primers. cDNA was diluted and expression of transcripts was determined using advanced qPCR master mix (Wisent Bioproducts). Fold change in transcript levels was determined using 2-delta Ct method (79) with normalization to the reference genes *HPRT1* or *ACTIN or B2M* (for primary mouse hepatocytes). The sequences of primers used in the study are listed in Table S4.

### Hepatocyte isolation and culture

Primary hepatocytes were isolated from 14 to 17-week-old female and male mice with the same genotypes used in our previous study (3, 4). All mice were on a C57BL/6J background. Animals were handled in accordance with the guidelines of the Canadian Council on Animal Care, and protocols were approved by the Animal Ethics Committee of Université Laval (CPAUL). The hepatocytes were isolated using the two-step collagenase perfusion method (80). Briefly, the liver was infused with 50 ml of perfusion buffer *via* the vena cava inferior followed by 50 ml of perfusion buffer containing (20 mg collagenase type II, 0.51 mM CaCl_2_, 137 mM NaCl, 7 mM KCl, 0.7 mM Na_2_HPO_4,_ and 10 mM Hepes). After the perfusion, the liver was excised and disintegrated in cell attachment medium (M199 medium supplemented with 10% FBS, 1% penicillin/streptomycin, 500 nM dexamethasone, 10 nM insulin, 200 nM thyroid hormone, 0.1% bovine serum albumin and 1× Glutamax). The cellular suspension was centrifuged at 400 rpm for 2 min. The cell viability was measured by trypan blue exclusion assay. The cells were resuspended in cell attachment media and seeded on collagen I pre-coated plates. After the culture medium was changed 2 h later for serum-free hepatocyte culture basal medium (M199 supplemented with 50× B27 supplement, 1× glutamax, 1% penicillin/streptomycin, 100 nM dexamethasone and 2 nM nicotinamide) to remove unattached cells, the hepatocytes were used for the experiments.

### Hepatic glucose production assay

Hepatic glucose production in FAO cells and PMH was essentially measured as described before (81). FAO cells expressing control shRNA or *Ptpn6* shRNA and PMH isolated from *Ptpn6*^*f/f*^ or *Ptpn6*^*H-KO*^ mice were serum-deprived overnight and either left untreated or treated with 50 μM Stat5 inhibitor (CAS 285986-31-4). Cells were washed three times with PBS and incubated with hepatic glucose production media (DMEM without glucose (Sigma-Aldrich #D5030-1L), containing sodium bicarbonate (3.7 g/l), sodium pyruvate (2 mM), and sodium L-lactate (20 mM), pH7.3) (with and without Stat5 inhibitor). After 5 h, supernatants were collected and glucose levels were determined using Amplex red Glucose/Glucose Oxidase assay kit (Invitrogen). Cells were washed with PBS and lysed in 50 mM NaOH. Protein levels were measured using the bicinchoninic acid assay method. Levels of glucose produced by the cells were normalized to the protein concentrations. Similarly, glucose production assay was performed in SHP-1 WT and SHP-1-KD FAO cells with or without STAT5-specific shRNA.

### Quantification and statistical analysis

All data are reported as means ± SD. Statistical analysis was performed using the GraphPad Prism 8 software (https://graphpad.com). We used an unpaired two-tailed *t* test or two-way ANOVA with Tukey’s post hoc test. The number of biological repeats included in the experiments is mentioned in the figure legends. Western blots were quantified using ImageJ.

### Materials availability

Further information and requests for resources and reagents should be directed to and will be fulfilled by the corresponding author, André Marette (Andre.Marette@criucpq.ulaval.ca).

## Data and code availability

The ChIP-seq data discussed in Figure 4 of this manuscript have been deposited in NCBI's Gene Expression Omnibus (82) and are accessible through GEO Series accession number GSE174142 (https://www.ncbi.nlm.nih.gov/geo/query/acc.cgi?acc=GSE174142).

## Supporting information

This article contains supporting information (83, 84, 85, 86).

## Conflict of interest

The authors declare that they have no conflicts of interest with the contents of this article.
