# Supplementary material for: SHP-1 phosphatase acts as a coactivator of PCK1 transcription to control gluconeogenesis
Source: J Biol Chem. 2023 Aug 16;299(9):105164. doi: 10.1016/j.jbc.2023.105164 (PMC10504565; doi:10.1016/j.jbc.2023.105164)
Supplement: Supporting information [file mmc3.docx]

**SHP-1 phosphatase acts as a co-activator of *PCK1* transcription to control gluconeogenesis**

Amit Kumar^1†^, Michael Schwab^1†^, Beisy Laborit Labrada^1^, Maruhen Amir Datsch Silveira^2,3,5^, Marilyn Goudreault^6,8^, Éric Fournier^2,3,4,5^, Kerstin Bellmann^1^, Nicole Beauchemin^7^, Anne-Claude Gingras^8,9^, Steve Bilodeau^2,3,4,5^, Mathieu Laplante^1,3^, and André Marette^1,10*^

^1^Centre de recherche de l'Institut universitaire de cardiologie et de pneumologie de Québec (CRIUCPQ), Faculté de Médecine, Université Laval, Québec, QC G1V 4G5, Canada.

^2^Centre de Recherche du CHU de Québec - Université Laval, Axe Oncologie, Québec, QC G1V 4G2, Canada.

^3^Centre de Recherche sur le Cancer de l’Université Laval, Québec, QC G1R 3S3, Canada.

^4^ Centre de recherche en données massives de l’Université Laval, Québec, QC G1V 0A6, Canada.

^5^Département de biologie moléculaire, biochimie médicale et pathologie, Faculté de Médecine, Université Laval, Québec, QC G1V 0A6, Canada.

^6^Institute for Research in Immunology and Cancer, Université de Montréal, Montréal, QC H3T 1J4, Canada.

^7^Rosalind and Morris Goodman Cancer Research Centre, Depts. of Oncology, Medicine and Biochemistry, McGill University, Montreal, QC H3A 1A3, Canada.

^8^Lunenfeld-Tanenbaum Research Institute, Mount Sinai Hospital, Sinai Health System, Toronto, ON M5G 1X5, Canada.

^9^Department of Molecular Genetics, University of Toronto, Toronto, ON M5S 1A8, Canada.

^10^Institute of Nutrition and Functional Foods, Laval University, Québec, QC G1V 0A6, Canada.

† These authors contributed equally to this work.

* To whom correspondence may be addressed. **Email:** [Andre.Marette@criucpq.ulaval.ca](mailto:Andre.Marette@criucpq.ulaval.ca)

Running title: Regulation of gluconeogenesis by SHP-1.

**SUPPLEMENTARY INFORMATION**

**Supplementary figures S1-S8**

**Supplementary Table S1 (separate excel file): SAINTexpress analysis related to Figure 1A.**

**Supplementary Table S2 (separated excel file): RPB1 signal densities in SHP-1 KO vs SHP-1 WT cells (related to Figure 4).**

**Table S3: Oligos used in the cloning of guide RNAs in pX459.**

**Table S4: Primer sequences used for RT-qPCR and ChIP-qPCR.**

**Table S5: shRNA sequences used in the study.**

**Table S6: List of the antibodies and reagents used in the study.**

**Table S7: Softwares and algorithms used in the study.**


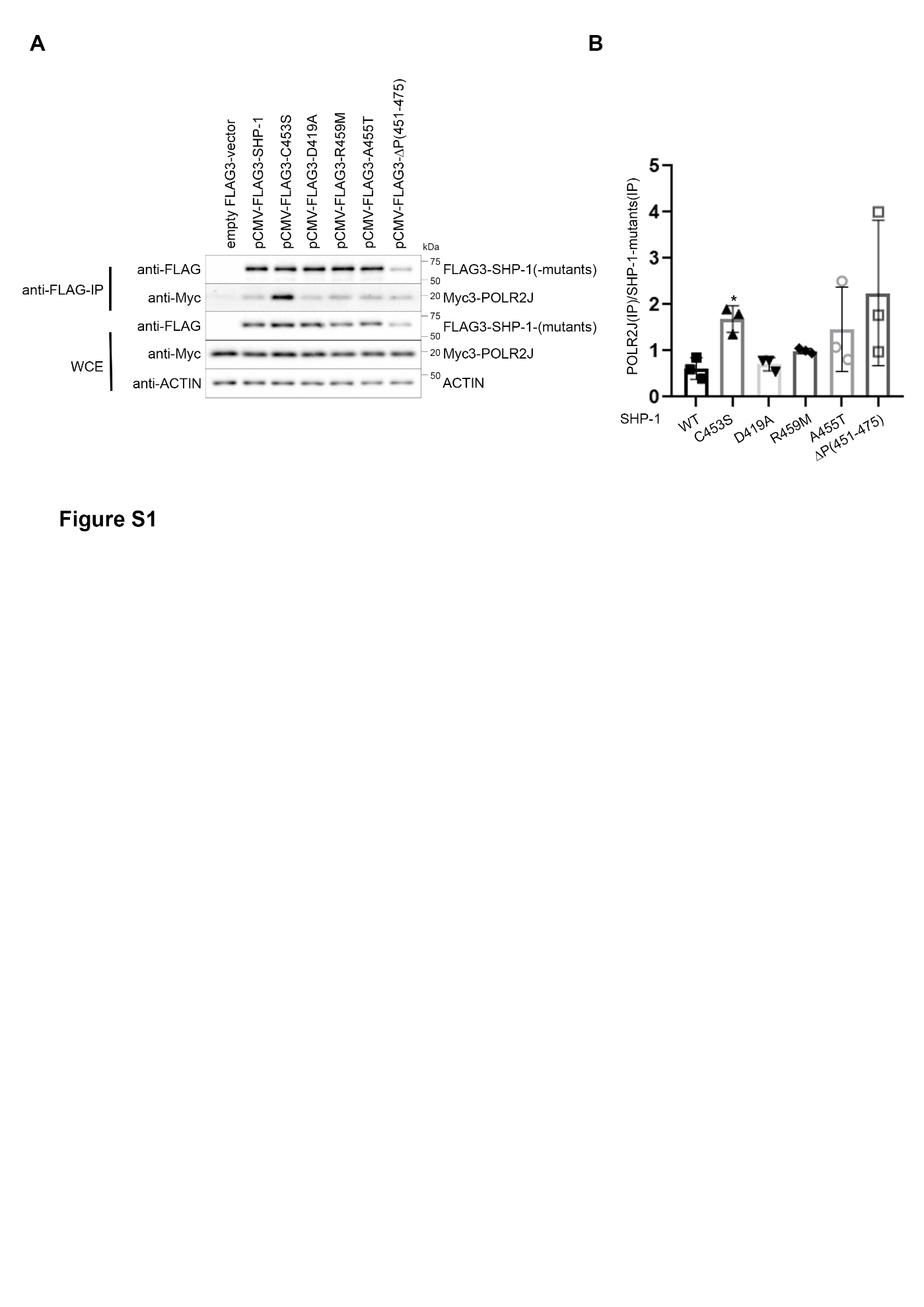


**Figure S1: POLR2J interacts differentially with catalytically compromised SHP-1 mutants.**

A. Western blot analysis of co-immunoprecipitations showing differential interaction of SHP-1-mutants with POLR2J in HepG2-cells co-expressing FLAG-tagged SHP-1-mutants and Myc-tagged POLR2J. Mutants: C453S = PTP-inactive, substrate-trapping, D419A = PTP-inactive, substrate-trapping, R459M = PTP-inactive, A455T = PTP less active, ΔP (Δ451-475) = PTP-inactive (deletion of active site).

B. Quantification of POLR2J co-immunoprecipitated with various SHP-1-mutants determined by densitometry using Image J (n=3). *p<0.05.


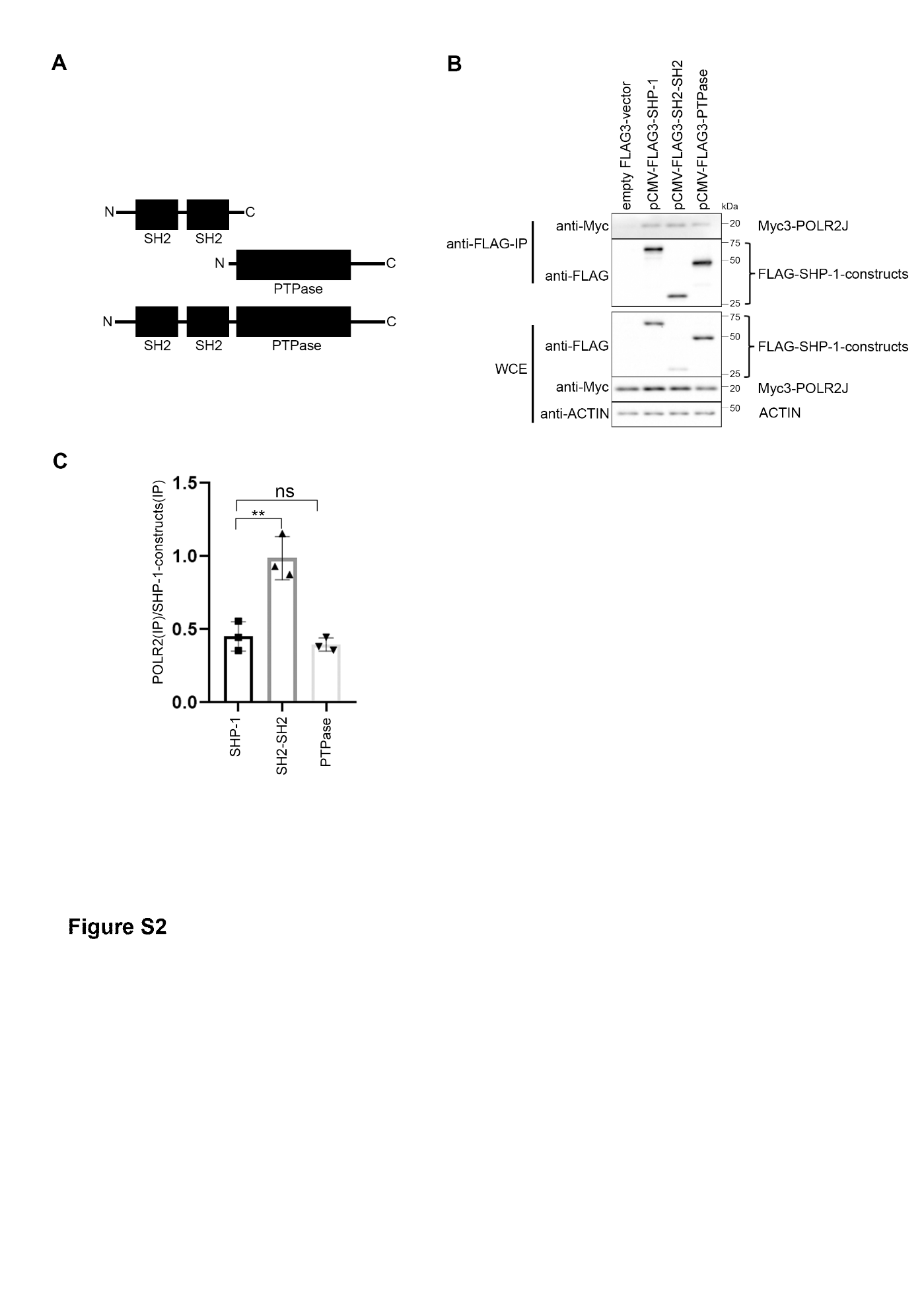


**Figure S2: SH2-domains of SHP-1 bind better to POLR2J than PTPase domain.**

A. Schematic of various SHP-1 constructs used in the study.

B. Western blot analysis of co-immunoprecipitations revealing differences in binding of SHP-1-(constructs) with POLR2J in HepG2-cells co-expressing FLAG-tagged SHP-1-constructs and Myc-tagged POLR2J.

C. Quantification of POLR2J co-immunoprecipitated with various SHP-1-constructs determined by densitometry using Image J (n=3). **p<0.01, ns = non-significant.


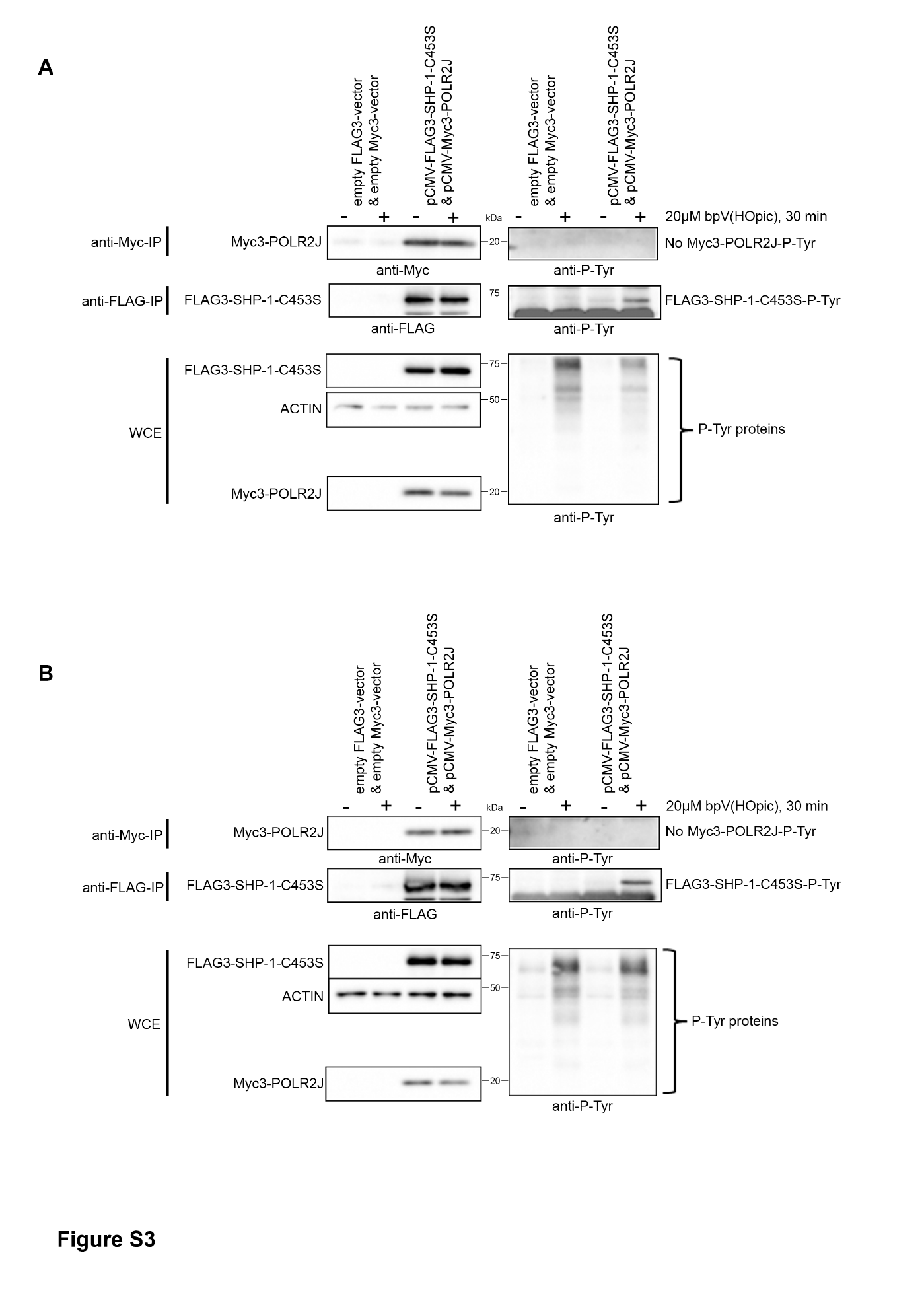


**Figure S3. SHP-1, but not POLR2J is tyrosine-phosphorylated.**

Western blot analysis of immunoprecipitations of Myc-tagged POLR2J and FLAG-tagged SHP-1-C453S showing no tyrosine-phosphorylation of POLR2J, but tyrosine-phosphorylation of SHP-1-C453S in Flp-In T-Rex 293- or HepG2-cells.

A. Flp-In T-Rex-cells containing either an empty FLAG3-vector or a FLAG3-SHP-1-C453S expression construct were transfected with an empty Myc3-vector or Myc3-POLR2J-expressing plasmid and treated with 20 μM bpV(HOpic) for 30 min or left untreated.

B. HepG2-cells were transfected with an empty FLAG3-vector or a FLAG3-SHP-1-C453S expression construct and an empty Myc3-vector or Myc3-POLR2J-expressing plasmid, respectively, and treated with 20 μM bpV(HOpic) for 30 min or left untreated.

Tyrosine-phosphorylation of Myc3-POLR2J and FLAG3-SHP-1-C453S precipitated with anti-Myc- and anti-FLAG-agarose beads was analyzed in immunoblots with phospho-tyrosine-specific antibodies.


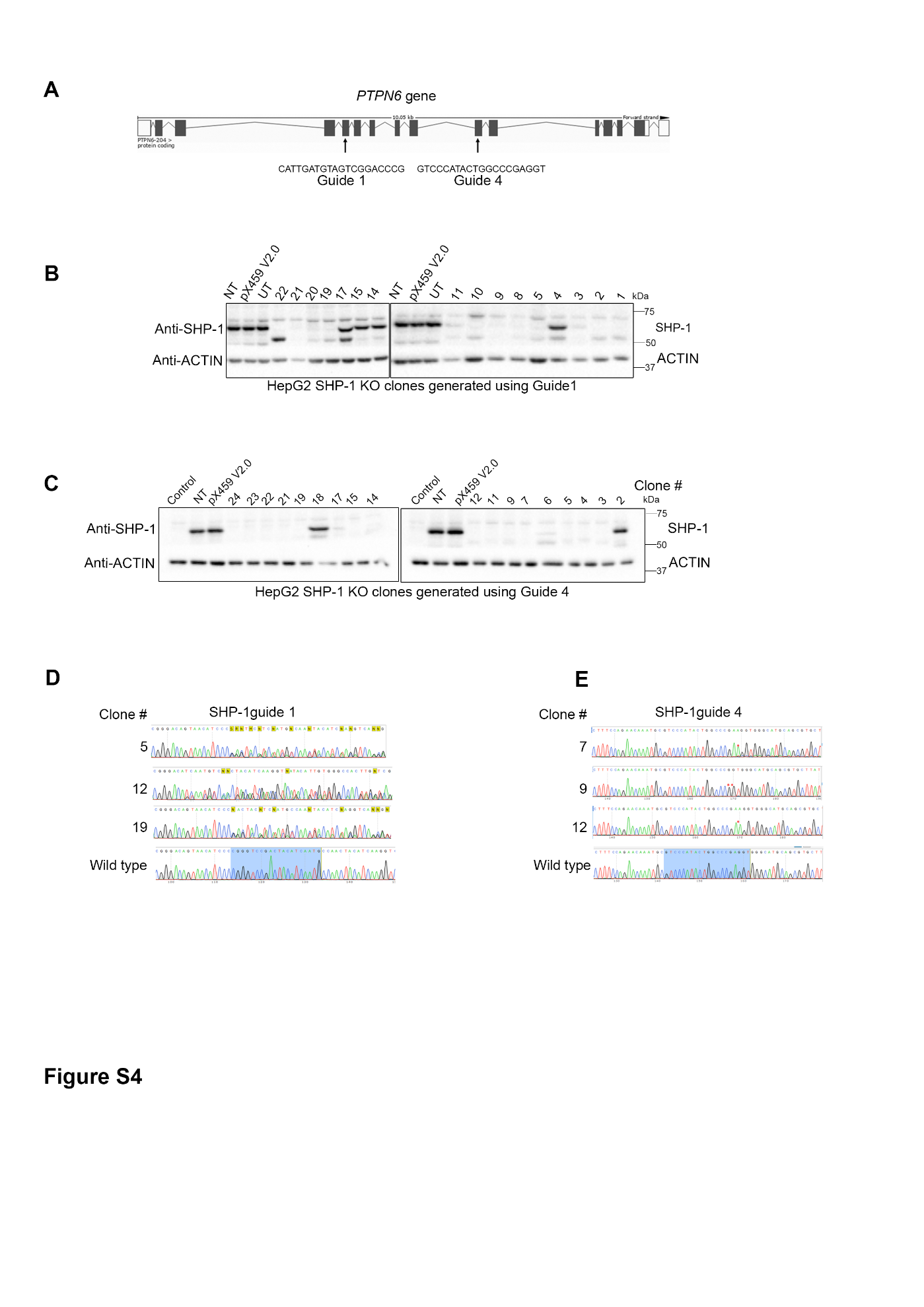


**Figure S4. Generation and validation of CRISPR mediated SHP-1 knockout HepG2 cells.**

HepG2 cells were transfected with pX459/SHP1 sgRNA #1 (A) or pX459/SHP sgRNA#4 (A) or pX459/non targeting sgRNA. Single cell clones were selected with puromycin. Expression of SHP-1 was determined by western blot using anti-Shp1 antibody (B & C). Sequencing chromatograms of genomic PCR products spanning single guide sites amplified from SHP-1 KO cells. Sequencing results of clones derived from sgRNA1 (D) and sgRNA4 (E) is shown.





**Figure S5. Knock down efficiency of shRNA in FAO cells.**

A. Confirmation by western blot of SHP-1 knockdown in FAO cells using lentiviral infection with constructs carrying luciferase-specific (control) or *SHP-1* (*Ptpn6*)-specific shRNA. Quantification of SHP-1 knockdown levels determined by densitometry using Image J (n=4). *p<0.05.

B. Confirmation by western blot of STAT5 knockdown in FAO cells (SHP-1-WT or SHP-1-KD) using lentiviral infection with constructs carrying either scramble control or *STAT5*-specific shRNA. Quantification of STAT5 knockdown levels determined by densitometry using Image J (n=2). *p<0.05, **p<0.01.





**Figure S6. Loss of SHP-1 coincides with reduction in *G6Pc* transcript levels.**

A. Expression levels of *G6Pc* transcripts in SHP-1 WT and KO HepG2 cells analyzed by qPCR. ***p <0.001 (n=3).

B. *G6pc*-mRNA levels in FAO cells with (*Ptpn6* shRNA) or without (control shRNA) knockdown of *SHP-1* determined by qPCR. *p<0.05 (n=2).

C. Levels of *G6pc* transcripts in primary hepatocytes isolated from *Ptpn6^f/f^* and *Ptpn6*^H-KO^ mice analyzed by qPCR. ***p<0.001 (n=3).

D. Levels of *G6pc* transcripts in liver lysates of *Ptpn6^f/f^* and *Ptpn6^H-KO^* mice analyzed by qPCR. ns = non-significant (n=5).


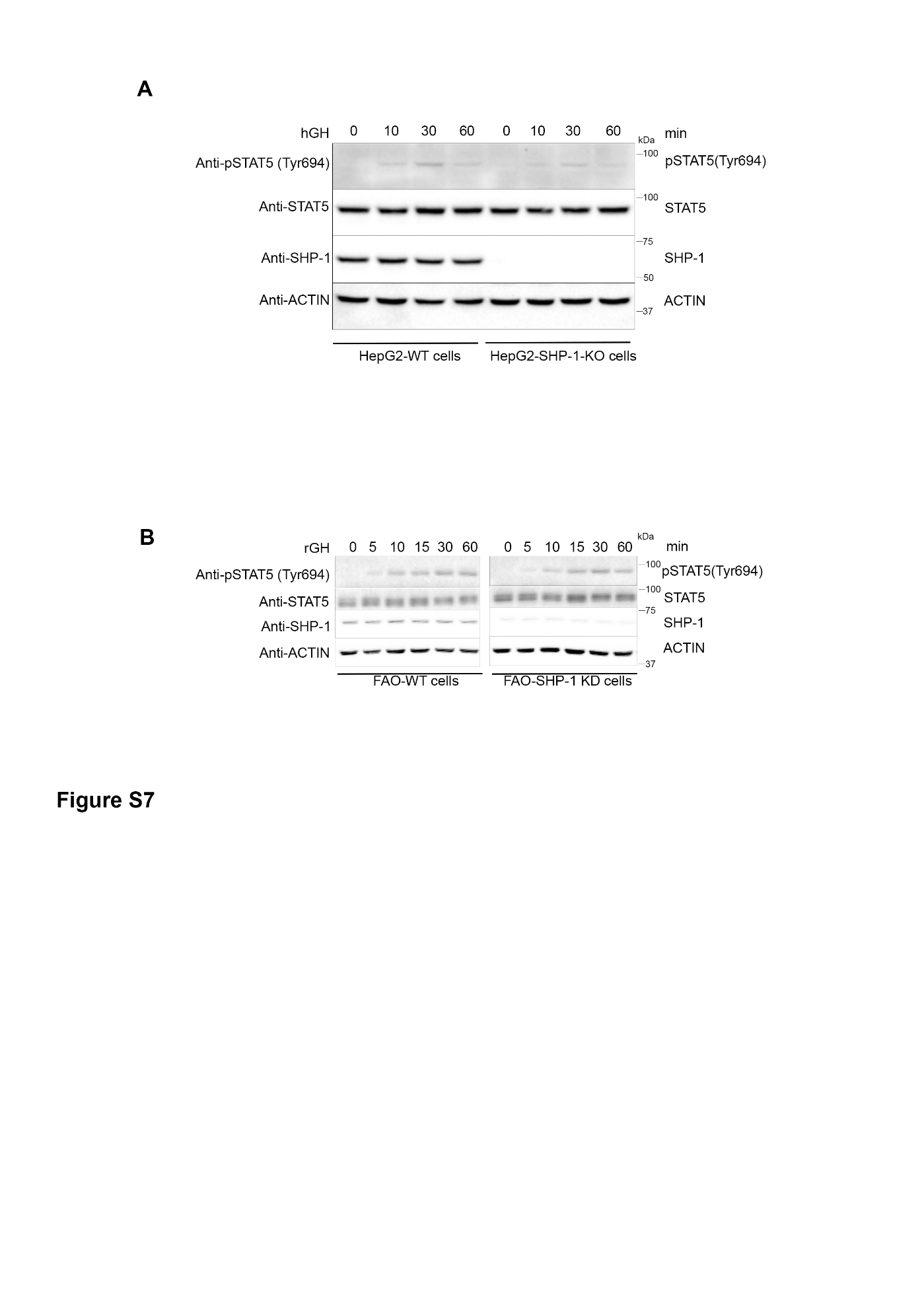


**Figure S7. STAT5-Y694 tyrosine-phosphorylation is not increased in SHP-1 KO cells.**

A. Western blot showing STAT5-Tyr694 phosphorylation and total amounts of various proteins in HepG2 SHP-1 WT or SHP-1 KO cells in response to recombinant human growth hormone treatment (500 ng/ml) at the indicated time points. (Representative of three experiments).

B. Western blot showing STAT5-Tyr694 phosphorylation and total amounts of various proteins in FAO cells with or without *SHP-1* knockdown in response to recombinant rat growth hormone treatment (500 ng/ml) at the indicated time points. (Representative of two experiments).





**Figure S8. Optimization of Chromatin immunoprecipitation PCR using Micrococcal Nucleases.**

A. Representative agarose gel analysis of Mnase digested chromatin.

B. Representative photographs of cross-linked cell pellets before and after Mnase digestion.

C. Efficiency of SHP-1 antibody in immunoprecipitation of chromatin fixed HepG2 cells analysed by western blotting.

| SgRNA | Species | Forward Primer | Reverse Primer |
| --- | --- | --- | --- |
| Sg1 | Human | CACC  GCATTGATGTAGTCGGACCCG | AAAC CGGGTCCGACTACATCAATGC |
| Sg4 | Human | CACC GTCCCATACTGGCCCGAGGT | AAAC ACCTCGGGCCAGTATGGGAC |
| Sg6 | Human | CACC GGGCCCGCATAGGATATCGC | AAAC GCGATATCCTATGCGGGCCC |

**Table S3: Oligos used in the cloning of guide RNAs in pX459.**

|  | Species | Forward Primer | Reverse Primer | Reference |
| --- | --- | --- | --- | --- |
| *Pck1* | Rat | TGCCCAAGATCTTCCACGTC | TCAAGTTCAGGGCGTCTTCC | (83) |
| *Actin* | Rat | CGTCTTCCCCTCCATCGT | GGAGTCCTTCTGACCCATACC | (83) |
| *PCK1* | Human | ATCTTTGGTGGCCGTAGACCT | CCGAAGTTGTAGCCGAAGAA | (84) |
| *HPRT1* | Human | AGATGGTCAAGGTCGCAAG | GTATTCATTATAGTCAAGGGCATATCC | (85) |
| *PCK1* | Mouse | GGCGATGACATTGCCTGGATGA | TGTCTTCACTGAGGTGCCAGGA | (86) |
| *G6pc* | Mouse | TCTACCTTGCTGCTCACTTTC | GGAGGCTGGCATTGTAGATG |  |
| *G6pc* | Human | GCATTAAACTCCTTTGGGTAGC | CGGAAGTGTTGCTGTAGTAGT |  |
| *G6pc* | Rat | GGACCTCCTGTGGACTTTGG | AAACGGAATGGGAGCGACTT |  |
| *B2M* | Mouse | GGGTGGAACTGTGTTACGTAG | TGGTCTTTCTGGTGCTTGTC |  |
| Chip*-PCK1* | Human | TGACCCACCTGCCTGTTAAG | ACTTCGAGCCCTCAACCAAC | Self designed |

**Table S4: Primer sequences used for RT-qPCR and ChIP-qPCR.**

**Table S5: shRNA sequence used in the study.**

| shRNA | Name | Sequence |
| --- | --- | --- |
| shShp1.68 | TRCN0000028968 | CCGGGCTAGACTGTGACATTGATATCTCGAGATATCAATGTCACAGTCTAGCTTTTT |
| shSTAT5A | TRCN0000232134 | GGACCTTCTTGTTGCGCTTTA |
|  |  |  |
| shSTAT5B | TRCN0000232140 | TATGTCCCTGAAACGAATTAA |
| shSTAT5B | TRCN0000012557 | GACTCTCAGGAGAGAATGTTT |
| STAT5B | TRCN0000421057 | GAATTTGCCAGGACGGAATTA |
| STAT5A | TRCN0000231567 | CGAGGTCTTTGCCAAGTATTA |
| STAT5A | TRCN0000231566 | GCCATTCACGACGCGAGATTT |

**Table S6: List of the antibodies and reagents used in the study.**

| Antibodies | Source | Identifier |
| --- | --- | --- |
| Mouse anti-SHP1 (1SH01) | ThermoFischer Scientific | MA5-11669 (1SH01) |
| Rabbit anti-SHP-1 (SH-PTP1) | Santa Cruz | sc-287 |
| Rabbit anti-POLR2J | Proteintech | 16403-1-AP |
| Rabbit anti-POLR2C | Proteintech | 13428-1-AP |
| Mouse anti-FLAG | Sigma | 088K6018 |
| Rabbit anti FLAG-tag | Proteintech | 20543-1-AP |
| Rabbt anti Myc-tag | Proteintech | 16286-1-AP |
| Mouse anti-MYC | Sigma | M4439-100UL |
| Mouse anti-ACTIN | Millipore | MAB1501 |
| Mouse anti-Phosphotyrosine (4G10) | Millipore | 05-321 |
| Mouse anti-Phosphotyrosine (PY20) | Abcam | Ab10321 |
| Rabbit anti-CREB | Cell Signaling | 9197S |
| Rabbit anti-Histone H3 | Santa Cruz | SC-10809 |
| Rabbit anti-GNPDA1 | Abcam | ab106563 |
| Rabbit anti-pSer2-RPB1  (anti-RNAPII-CTD-pSer2) | Abcam | ab5095 |
| Mouse anti-RPB1  (anti-RNAPII-CTD) | Abcam | ab817 |
| Rabbit anti-V5 | Cell Signaling | 13202S |
| Mouse anti-Phospho STAT5 (Tyr694) (14H2) | Cell Signaling | 9356 |
| Rabbit anti-STAT5  Rabbit anti eEF2 | New England Biolabs  Cell Signaling | 94205S  2332 |
| Anti-FLAG® M2 Magnetic Beads | Sigma | M8823-5ML |
| Anti-FLAG® M2 Affinity Gel  Anti-c-Myc Agarose Conjugate  Mouse IgG2b | Sigma  Sigma  Biolegend | A2220-5ML  A7470  MG2b-57 |
| Reagents |  |  |
| Tetracycline | Sigma | T7660-5G |
| Blasticidin S HCl | Invitrogen | R210-01 |
| Hygromycin B  Zeocin  Puromycin dihydrochloride | Invitrogen  Invitrogen  Invivogen | 10687-010  R250-01  ant-pr-1 |
| Insulin (Humulin R)  Poly-L-lysine hydrobromide | Eli Lilly  Sigma | HI0213  P1399-25MG |
| STAT5 inhibitor | Sigma | 573108-10MG-M |
| DMEM low glucose | Wisent Bioproducts | 319-010-CL |
| DMEM high glucose | Wisent Bioproducts | 319-005-CL |
| RPMI | Wisent Bioproducts | 350-000-CL |
| Jet Prime | Polyplus-transfection | 114-15 |
| PureFection  Dynabeads protein G | System Biosciences  Thermo Fisher Scientific | LV750A-1  10003D |
| Sodium orthovanadate | Sigma | S6508-50G |
| Sodium Fluoride | Sigma | S6776-100G |
| Calcium chloride | Sigma | C3981-500G |
| Sodium dodecyl sulfate | Sigma | L3771-500G |
| EDTA | Sigma | 60-00-4 |
| EGTA | Sigma | 03777 |
| β-glycerophosphate | Sigma | G5422-100G |
| Triton | Fisher | X-100 |
| SDS | Sigma | L3771-500G |
| dexamethasone | Sigma | D-1756 |
| BSA | Sigma | A9647-100G |
| Glutamax | Gibco | 35050-061 |
| FBS | Gibco | 16170-078 |
| RNase A | Invitrogen | 12091021 |
| Proteinase K | Invitrogen | 25530049 |
| Micrococcal Nuclease​ | New England Biolabs | M0247S |
| Advanced qpcr mastermix with supergreen lo-rox | Wisent bioproducts | 800-431-UL |
| Subcellular Protein Fractionation Kit for Cultured Cells | Thermo Fischer Scientific | 78840 |
| QIAquick PCR Purification Kit | Qiagen | 28104 |
| Amplex™ Red Glucose/Glucose Oxidase Assay Kit | Invitrogen | A22189 |

**Table S7: Softwares and algorithms used in the study.**

| Software | Source | Identifier |
| --- | --- | --- |
| Generunner | Hastings Software. Inc. Hastings, NY, USA | www.generunner.net |
| BioGRID | (35) | thebiogrid.org |
| PROMO 3.02 | (34) | http://alggen.lsi.upc.es/cgi-bin/promo_v3/promo/promoinit.cgi?dirDB=TF_8.3 |
| Venn Diagram | Bioinformatics & Evolutionary Genomics, BELGIUM | http://bioinformatics.psb.ugent.be/webtools/Venn/ |
| UCSC browser | (76) | <https://genome.ucsc.edu/> |
| CRISPR design tool | (70) | http://crispr.mit.edu/ |
|  |  |  |
